# G-quadruplexes Stabilization Upregulates CCN1 and Accelerates Aging in Cultured Cerebral Endothelial Cells

**DOI:** 10.3389/fragi.2021.797562

**Published:** 2022-01-12

**Authors:** Brian Noh, Maria P. Blasco-Conesa, Yun-Ju Lai, Bhanu Priya Ganesh, Akihiko Urayama, Ines Moreno-Gonzalez, Sean P. Marrelli, Louise D. McCullough, Jose Felix Moruno-Manchon

**Affiliations:** ^1^ Department of Neurology, McGovern Medical School at the University of Texas Health Science Center at Houston, Houston, TX, United States; ^2^ Solomont School of Nursing, Zuckerberg College of Health Sciences, University of Massachusetts Lowell, Lowell, MA, United States; ^3^ Department of Cell Biology, Faculty of Sciences, Instituto de Investigacion Biomedica de Malaga-IBIMA, Malaga University, Malaga, Spain; ^4^ Networking Biomedical Research Networking Center on Neurodegenerative Diseases (CIBERNED), Madrid, Spain

**Keywords:** senescence, endothelial cells, G-quadruplex, aging, CCN1

## Abstract

Senescence in the cerebral endothelium has been proposed as a mechanism that can drive dysfunction of the cerebral vasculature, which precedes vascular dementia. Cysteine-rich angiogenic inducer 61 (Cyr61/CCN1) is a matricellular protein secreted by cerebral endothelial cells (CEC). CCN1 induces senescence in fibroblasts. However, whether CCN1 contributes to senescence in CEC and how this is regulated requires further study. Aging has been associated with the formation of four-stranded Guanine-quadruplexes (G4s) in G-rich motifs of DNA and RNA. Stabilization of the G4 structures regulates transcription and translation either by upregulation or downregulation depending on the gene target. Previously, we showed that aged mice treated with a G4-stabilizing compound had enhanced senescence-associated (SA) phenotypes in their brains, and these mice exhibited enhanced cognitive deficits. A sequence in the 3′-UTR of the human *CCN1* mRNA has the ability to fold into G4s *in vitro*. We hypothesize that G4 stabilization regulates CCN1 in cultured primary CEC and induces endothelial senescence. We used cerebral microvessel fractions and cultured primary CEC from young (4-months old, m/o) and aged (18-m/o) mice to determine CCN1 levels. SA phenotypes were determined by high-resolution fluorescence microscopy in cultured primary CEC, and we used Thioflavin T to recognize RNA-G4s for fluorescence spectra. We found that cultured CEC from aged mice exhibited enhanced levels of SA phenotypes, and higher levels of CCN1 and G4 stabilization. In cultured CEC, CCN1 induced SA phenotypes, such as SA β-galactosidase activity, and double-strand DNA damage. Furthermore, CCN1 levels were upregulated by a G4 ligand, and a G-rich motif in the 3′-UTR of the *Ccn1* mRNA was folded into a G4. In conclusion, we demonstrate that CCN1 can induce senescence in cultured primary CEC, and we provide evidence that G4 stabilization is a novel mechanism regulating the SASP component CCN1.

## Introduction

The blood-brain barrier (BBB) is the physical barrier between the brain parenchyma and the periphery (Wang et al., 2018). It is formed by cerebral endothelial cells (CEC), pericytes, astrocytes, and smooth muscle cells. A healthy cerebrovascular endothelium is essential to maintain low and selective permeability of the BBB (Chen and Liu 2012), which allows both proper nutrient delivery to the brain and protection from infections. However, dysfunction of CEC can contribute to BBB disruption (Jia et al., 2019). Thus, identifying molecular mechanisms that can promote endothelial damage is critical for our understanding of cerebral endothelium dysfunction, and to guide us in developing new therapeutic strategies to preserve BBB function.

During aging, CEC enter into a non-proliferative state named senescence. This state is characterized by enhanced DNA damage (Lahteenvuo and Rosenzweig 2012) and secretion of pro-inflammatory cytokines that induce senescence in neighboring cells (Tchkonia et al., 2013). This “senescent-associated secretory phenotype (SASP)” in CEC contributes to cerebral endothelium dysfunction and dementia (Wang et al., 2018; Graves and Baker 2020). This senescence phenotype adversely affects the cerebral vasculature in a multifaceted manner (Pelegri et al., 2007; Del Valle et al., 2009; Yamazaki et al., 2016; Graves and Baker 2020). Currently, the agents that induce CEC senescence are poorly defined.

The extracellular matrix protein cysteine-rich angiogenic inducer 61 (Cyr61/CCN1) participates in a wide variety of functions, such as promoting cell survival, cell proliferation, angiogenesis and wound repair. However, CCN1 has also been shown to promote senescence in fibroblasts (Jun and Lau 2010) and is one of the characteristic SASP genes. In the brain, CCN1 is produced and secreted by endothelial cells and astrocytes. Recently, it was found that *Ccn1* is the most highly upregulated gene in senescence-induced astrocytes, but not in reactive astrocytes (Simmnacher et al., 2020), highlighting its specific role in cellular senescence. Whether the SASP factor CCN1 contributes to senescence specifically in CEC and how CCN1 is regulated warrants further study.

Aging is associated with the formation of four-stranded Guanine-quadruplexes (G4s) in guanine-rich motifs of DNA and RNA (Lejault et al., 2020; Moruno-Manchon et al., 2020; Teng et al., 2020). Hoogsteen hydrogen bonds align four guanines in a sequence in a planar tetrad, and several tetrads can be stacked in one single G4. Growing evidence in living cells indicates that G4 stabilization regulates transcription (Siddiqui-Jain et al., 2002; Agarwal et al., 2014; Armas et al., 2017; Moruno-Manchon et al., 2017; Lejault et al., 2020; Moruno-Manchon et al., 2020) and translation (Schaeffer et al., 2001; Khateb et al., 2007), either upregulating or downregulating expression depending on the gene target (Armas et al., 2017; Fay et al., 2017). G4s are also involved in RNA splicing (Zhang et al., 2019), mRNA subcellular localization (Subramanian et al., 2011), and DNA integrity (Kruisselbrink et al., 2008; Castillo Bosch et al., 2014; Moruno-Manchon et al., 2017; Techer et al., 2017). Previously, we found that treating aged mice with a G4-stabilizing compound led to enhanced SA phenotypes in the brain (DNA damage and lipofuscin accumulation), compared with age-matched controls (Moruno-Manchon et al., 2020). Mice treated with a G4-stabilizer also exhibited enhanced cognitive deficits in comparison to control mice (Moruno-Manchon et al., 2020). We further demonstrated that stabilizing G4s induced DNA damage in cultured neurons (Moruno-Manchon et al., 2017) and contributed to impairment of autophagy, which is associated with senescence (Moreno-Blas et al., 2019; Salazar et al., 2020; Sunderland et al., 2020). In addition, G4 stabilization induced DNA damage in cultured astrocytes and microglia (Lejault et al., 2020; Moruno-Manchon et al., 2020; Tabor et al., 2021). Thus, the stability of G4 structures appears to have a wide influence in cells relevant to the development of dementia.

In this study, we show that CCN1 is upregulated in cultured primary CEC derived from aged mice compared with those derived from young mice. Treating CEC with a recombinant CCN1 protein exacerbated SA phenotypes in cultured CEC from aged mice. CCN1 levels were upregulated by a G4 stabilizing drug, which promoted CEC senescence. Finally, we propose that CCN1 levels increase by the stabilization of G4 in a motif found in the 3′-UTR of the *Ccn1* mRNA. Thus, our study highlights the role of CCN1 in contributing to endothelial senescence and proposes a novel mechanism by which SASP component CCN1 is regulated.

## Materials and Methods

### Animals and Ethics Statement

3–4 and 18–20 months old (m/o) C57BL/6J female mice were obtained from Jackson laboratories and raised in the animal facilities at the University of Texas McGovern Medical School. The experiments were conducted following the protocol approved by the Center for Laboratory Medicine and Care (CLAMC, protocol number AWC-21-0084) at the University of Texas McGovern Medical School. Mice were maintained in an environment with constant temperature and humidity on a 12 h light/12 h dark schedule, and with *ad libitum* access to water and mouse lab pellets. Researchers and veterinarians manipulated mice with no distress, pain, or injury.

### Antibodies and Reagents

Antibodies against CCN1 were obtained from Santa Cruz Technologies (#sc-374129). Antibodies against p16^INK4A^ were from Biomatik (#CAF10165). Antibodies against BG4 were from Sigma-Millipore (#MABE917). Antibodies against CD31 (#ab28364), γH2AX (#ab11174), and β-Tubulin (#ab7751) were from Abcam.

Recombinant Cyr61/CCN1 protein was from Novus Biologicals (#NBP2-34944). Pyridostatin was purchased from Cayman (#18013). Thioflavin T was purchased from Sigma-Aldrich (#T3516). RNA oligos were purchased from Sigma-Millipore (#VC40001). Hoechst dye was from Santa Cruz Biotechnology (#SC-394039).

### Isolation of Cerebral Endothelial Cells From Adult Mouse Brains and Culture

Primary CEC were isolated from adult 3–4 and 18–20 m/o old C57BL/6J mice using the Adult Brain Dissociation Kit according to the manufacturer’s instructions (MACS Miltenyi Biotec, #130-107–677). Briefly, mouse brains were homogenized and enzymatically digested. Homogenized brains were filtered through 70 µm-pore strainers, and debris was separated by centrifugation (3,000 × *g*, 10 m). Cellular suspensions were then treated to remove red blood cells. Finally, cellular suspension was plated in pre-coated flasks with 0.1% sterile porcine gelatin (Biological Industries, #01-944-1B). Cells were maintained in Complete Mouse Endothelial Cell Medium/w Kit (Cell Biologics, #M1168) supplemented with puromycin (4 μg/ml) for 48 h to select endothelial cells (Perriere et al., 2005). After 48 h, cells were washed with PBS to remove puromycin and debris. Cells were then maintained in fresh Complete Mouse Endothelial Cell Medium until 100% confluence.

### Isolation of Cerebral Microvessels From Adult Mice

Mouse brains were dissected and the cortices were homogenized in MCDB 131 medium (ThermoFisher, #14190144) using a loose-fit 7-ml Dounce tissue grinder and centrifuged (2,000 × *g*, 2 m). After centrifugation, the pellets were resuspended in 15% (wt/vol) dextran–DPBS and centrifuged (10,000 × *g*, 15 m) to remove white matter. The pellets were again resuspended with DPBS and filtered through 40 µm strainer. Microvessels remained in the filter and were fixed with 4% paraformaldehyde-DPBS for 10 m. The strainer was inverted and microvessels were collected in a 50 ml tube with DPBS and centrifuged. The pellets were resuspended. 50 µl of microvessels suspensions were placed on a microscope slide and air-dried overnight for subsequent immunostaining (Lee et al., 2019).

### Immunocytochemistry

Cultured CEC were fixed with 4% paraformaldehyde for 10 m, washed with PBS, and permeabilized with a 0.5% Triton-X100/PBS solution for 10 m at room temperature. Fixed cells or microvessels were blocked with a solution containing 5% bovine serum albumin and 0.1% Triton X-100 in PBS, at room temperature for 1 h. Then, cells or microvessels were incubated with a solution of primary antibodies in blocking solution overnight at 4°C. Cells or microvessels were washed and incubated with Alexa Fluor®-conjugated secondary antibodies against the host species used to generate the primary antibodies (Abcam), and with the Hoechst dye to stain nuclei, which was imaged in the DAPI channel.

A blinded investigator imaged 5–10 microscopic fields (×20 and ×40 objectives) using the same exposure time and light intensity. We established threshold limits for each marker for quantitative analyses. The mean of fluorescence intensities in each region of interest was quantified with ImageJ software. The fluorescence intensities from the background of each picture were subtracted from the correspondent values of the region of interest in the same image.

### Western Blotting

Cultured primary CEC were collected in RIPA buffer (150 mM NaCl, 1% Nonidet P40, 0.5% sodium deoxycholate, 0.1% SDS and 50 mM Tris/HCl pH 8.0), with a cocktail of phosphatase and protease inhibitors (Sigma Aldrich). Cells were lysed with three cycles of freezing-thawing. Each cycle consisted of 5 m in dry ice, 30 s in a water bath at 37°C, 5 m on ice, and vortex samples for 30 s. Cellular lysates were cleared by centrifugation at 15,000 × *g* for 15 m at 4°C. The supernatants were collected and protein concentrations were analyzed by the PierceTM Bicinchoninic Acid Protein Assay Kit (ThermoFisher Scientific, #23225). Cleared lysates were diluted in sample buffer, boiled at 95°C and run in 4–20% Mini-PROTEAN TGX gels (BioRad). Proteins were transferred on to nitrocellulose membranes in transfer buffer containing 20% methanol at 0.2 Å for 2 h at 4°C. Membranes were blocked with 5% non-fat dry milk (1 h at room temperature). Membranes were incubated with primary antibodies overnight at 4°C. Then, membranes were incubated with secondary antibodies against the host species used to generate the primary antibodies conjugated with HRP 1 h at room temperature. Chemiluminescent signal was produced using SuperSignal™ West Femto Maximum Sensitivity Substrate (ThermoFisher, #34094) and detected with a charge-coupled device camera-based scanner.

The band intensity from each lane of the protein of interest was quantified with the ImageJ software and related to the band intensity of the correspondent lane of β-Tubulin.

### Senescence-Associated β-galactosidase Staining

Cultured CEC were fixed with 4% paraformaldehyde at room temperature for 5 m, washed with PBS, and incubated with the β-galactosidase Staining Solution (1 mg/ml X-Gal, pH 6.0) from the Senescence β-galactosidase Staining Kit (Cell Signaling, #9860) at 37°C, accordingly to the manufacturer’s instructions. Fixed cells from all experiments were incubated for the same time (24 h) to avoid intensity changes due to differences in incubation times. The percentage of cells positive to the Senescence β-galactosidase Staining was calculated by a blinded investigator to experimental groups using ImageJ software.

### RNA G4 Detection Using Thioflavin T

The oligonucleotides corresponding to the 3′-UTR of *Ccn1* mRNA and a negative control (G > T in the putative G4 forming region) were dissolved in a buffer (40 mM KCl, 20 mM tris-HCl, pH 7.0) for a 100 µM stock solution. Thioflavin T was dissolved in hot water for a 100 µM stock solution. The RNA stocks were dissolved in the buffer (4 µM) and annealed by heating at 90°C for 2 m and then cooled down slowly to room temperature for 2 h. The annealed RNA samples, or only the buffer as control, were mixed with Thioflavin T (2 µM) in a 96-well plate and the fluorescence emission was measured at 484 nm with excitation at 440 nm in a microplate reader (Xu et al., 2016). The oligonucleotide sequences are:

3′-UTR *Ccn1* –G1: UCA​UGG​AGA​CGU​GGG​UGG​GCG​GAG​GAU​GAA​U.

3′-UTR *Ccn1* –G1 Negative Control: UCA​UGUAGA​CGU​GGG​UGG​GCG​GAG​GAU​GAA​U.

### Gene Expression by RT-qPCR

Cultured CEC were collected and processed following the manufacturer’s instructions of the RNeasy Mini Kit (Qiagen, #74104) to isolate total RNA. RNA was reverse transcribed with the iScript Reverse Transcription SuperMix (Bio-Rad, #1708840) and then used to analyze the gene expression by qPCR. 2 µl of cDNA of a sample were diluted in iTaq Universal SYBR^®^ Green Supermix (Bio-Rad, #1725121) per reaction and run using a Bio-Rad CFX96 Touch device (95°C for 3 m, and 40 cycles of 95°C for 10 s and 55°C for 30 s).

Sequences of primers: Forward Mouse *Ccn1* 5′-GTG​AAG​TGC​GTC​CTT​GTG​GAC​A-3′; Reverse Mouse *Ccn1* 5′-CTT​GAC​ACT​GGA​GCA​TCC​TGC​A-3′; Forward Mouse *Gapdh* 5′-CAA​GGT​CAT​CCA​TGA​CAA​CTT​TG-3′; Reverse Mouse *Gapdh* 5′-GTC​CAC​CAC​CCT​GTT​GCT​GTA​G-3′. The relative expression of the gene of interest was calculated with the double delta Ct method related to the relative expression of *Gapdh*.

### RNA Sequencing

Cultured CEC isolated from young and aged male mice were cultured and maintained in 75 cm^2^ flask until cells reached 100% confluence. mRNA from each flask was isolated using the RNeasy Mini kit (Qiagen, #74104) and reverse transcribed with the iScript cDNA Synthesis kit (Bio-Rad, #1708890). cDNAs were sent to Qiagen for RNA sequencing analyses.

### Bioinformatics Analysis

Putative G4 motifs in the *Ccn1* mRNA were predicted using the G4Hunter bioinformatics tool (http://bioinformatics.ibp.cz/#/) with the following settings: Threshold = 1.2; Window size = 25. A threshold of 1.2 provides a good estimation of potential G4 forming motifs with a false discovery rate (FDR) of 10%. Window size of 25 is closed to the mean length of G4 forming sequences (26 nucleotides) (Bedrat et al., 2016). The G4Hunter analyses give a score for each analyzed sequence. A G4Hunter score of a sequence above 1 is regarded as a good predictor of its ability to fold into a G4 *in vitro* (Bedrat et al., 2016).

### Statistics

All analyses were performed using GraphPad Prism software (v.7). We used Student’s t-test to compare means from two independent groups. A value of *p* < 0.05 was considered significantly different. Bar graphs represent mean ± SEM.

## Results

### Cultured Primary CEC Isolated From the Brains of Aged Mice Show Senescence-Associated Phenotypes Compared With Young Mice

We first evaluated the purity of our primary CEC culture preparation. Cultures of primary CEC from adult mice showed minimal contamination with other brain cell types (Supplementary Figure S1). We compared the transcriptional signature of our CEC culture obtained from RNA sequencing analysis with that from the microvasculature of mice (Song et al., 2020), and found that 41% of the 1,000 highest upregulated genes found in cultured CEC from young males corresponded with the most upregulated genes found in the microvessels of 6–7 weeks old male mice (Supplementary Table S1). These genes are involved in important endothelial functions such as, transcription, translation, transport, cell-cell interaction, metabolism, proliferation, exocytosis and endocytosis, etc. Thus, our primary culture of CEC conserves important features of the brain vasculature and is relevant to studies of the cerebral endothelium.

**FIGURE 1:**
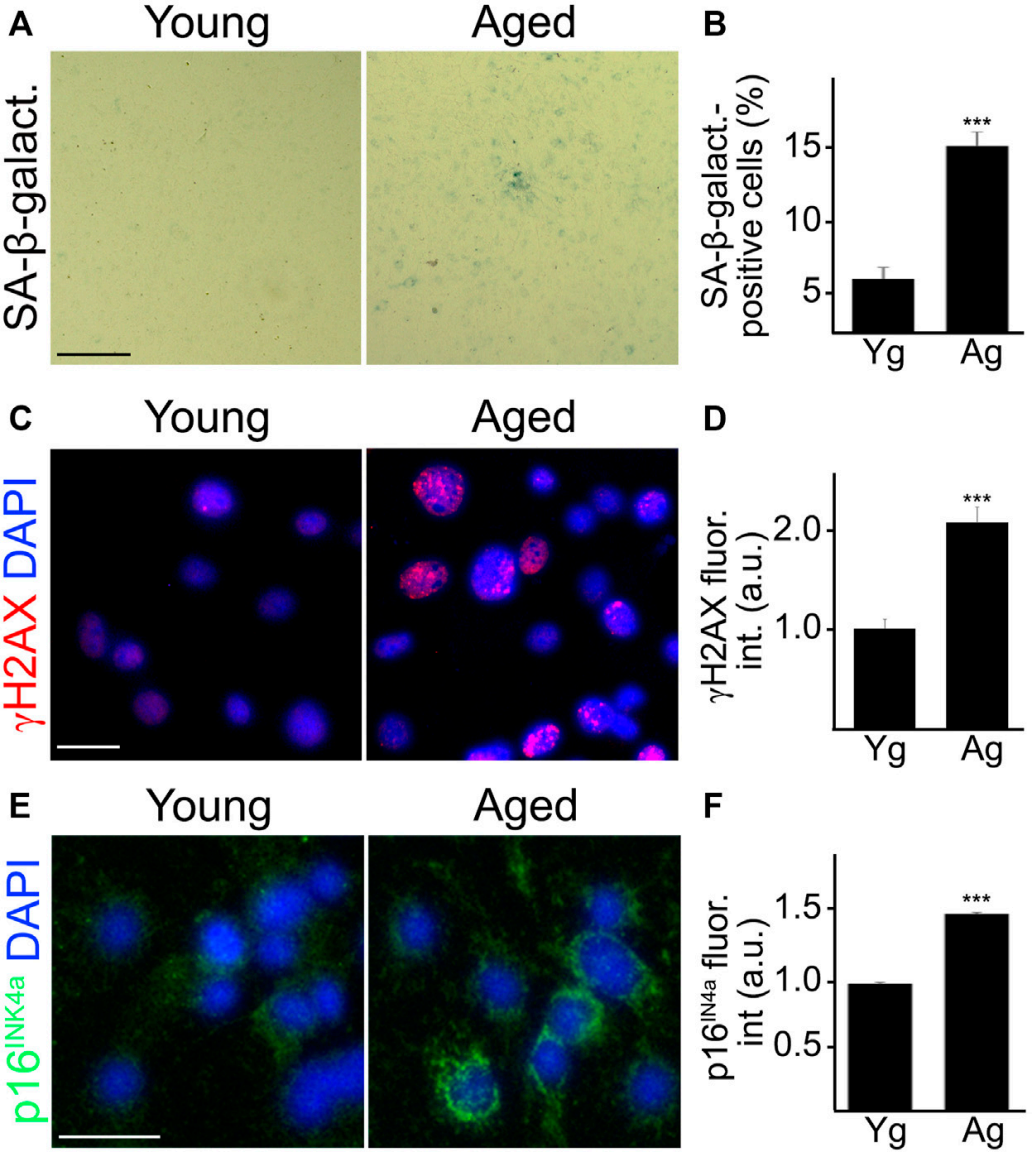
Cultured primary CEC from aged mice recapitulated senescence-associated phenotypes observed *in vivo*. CEC were isolated from young (3–4 m/o) and aged (18–20 m/o) female mice and cultured. Young and aged cultured CEC were stained for SA-β-galactosidase **(A)**, with antibodies against the double-strand DNA damage marker γH2AX **(C)**, or with antibodies against the tumor suppressor p16^INK4A^ **(E)**. The nuclear Hoechst dye was used to stain nuclei and imaged with the DAPI channel **(C**,**E)**. The percentage of SA-β-galactosidase-positive cells **(B)**, the fluorescence intensity of γH2AX **(D)** and the fluorescence intensity of p16^INK4A^ **(F)** were quantified. Scale bar **(A)**, 100 μm; scale bar **(C)**, 25 μm; scale bar **(E)**, 25 μm. *t*-test, ***p-value < 0.0001. Data were pooled from three independent experiments, 20 microscopic fields **(A**,**B)**, or 50 cells **(C**–**F)** per each experiment and condition.

We next generated primary CEC from the brains of 4 and 18-m/o mice to evaluate SA-phenotype. SA-β-galactosidase staining is commonly used as a marker of senescent cells as these cells exhibit enhanced activity of the lysosomal enzyme SA-β-galactosidase (Dimri et al., 1995). Thus, CEC were stained for SA-β-galactosidase, and quantified as the percentage of cells positive to SA-β-galactosidase (Figures 1A,B). In addition, CEC from young or aged mice were stained with antibodies against γH2AX and p16^INK4a^. γH2AX is a marker of double-strand breaks, the most severe form of DNA damage (Noubissi et al., 2021); and p16^INK4a^ is a tumor suppressor, overexpression of which is associated with cell cycle arrest and cell senescence (Oh et al., 2021). Aged CEC exhibited higher levels of both the DNA damage marker γH2AX (Figures 1C,D) and the tumor suppressor p16^INK4a^ (Figures 1E,F), compared with young cells. These findings indicate that the age-dependent senescent phenotype is recapitulated in the primary CEC, and that this experimental approach can be useful in the study of cerebral endothelial aging.

### Cultured CEC Isolated From Aged Mice Exhibit Higher Levels of G4

G4 stabilization has been associated with aging (Moruno-Manchon et al., 2017; Lejault et al., 2020; Moruno-Manchon et al., 2020; Teng et al., 2020). Previously, we found that treating aged mice with pyridostatin (PDS) induced DNA damage, lipofuscin accumulation, and memory impairment in aged mice (Moruno-Manchon et al., 2020), indicating that the G4 structures play a causative role in SA phenotypes and aging.

**FIGURE 2:**
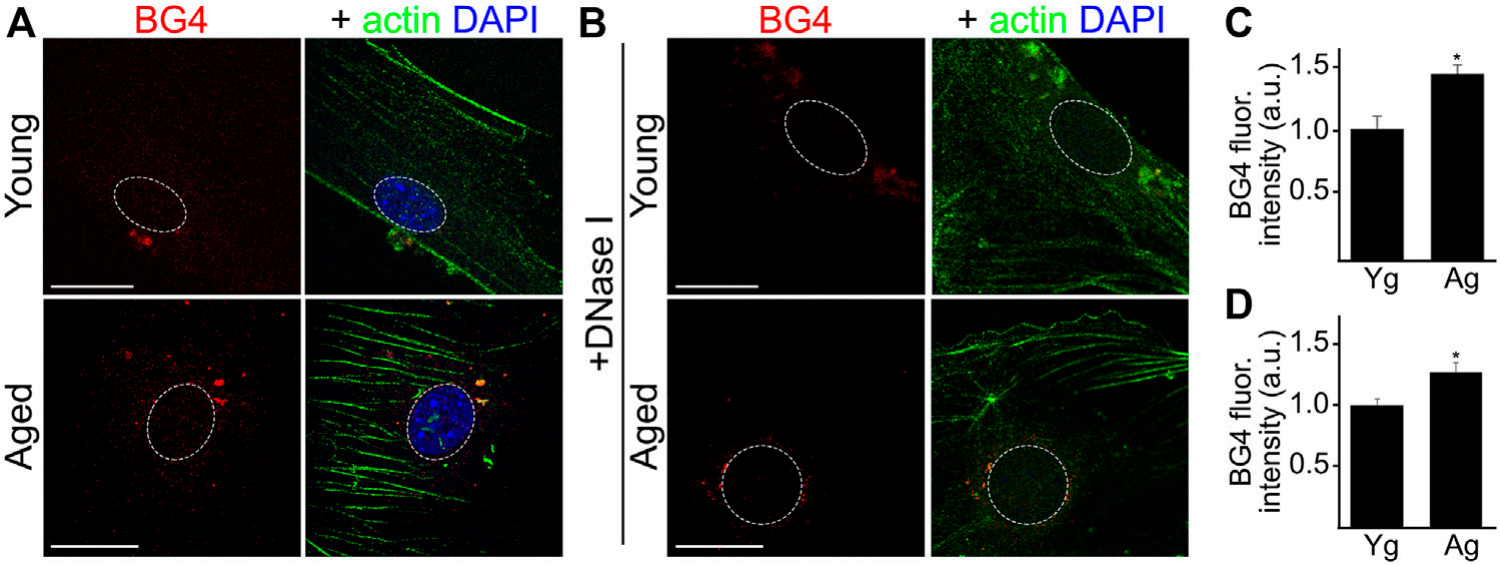
Cultured CEC from aged mice showed higher levels of RNA-G4 than cultured young CEC. **(A)** CEC were isolated from young (4-m/o) and aged (20-m/o) female mice and cultured. Cells were fixed and stained with antibodies against G4 (BG4) anti against actin, and with the nuclear Hoechst dye. **(B)** Cultured CEC from young and aged female mice were fixed and incubated with DNaseI to eliminate DNA G4, and then stained with antibodies against G4 (BG4) and against actin, and with the nuclear Hoechst dye. **(C**,**D)** BG4 fluorescence intensities were measured. **(C)** Quantification from **(A)**. Scale bar (Yg), 20 μm; Scale bar (Ag), 40 μm. *t*-test, *p-value = 0.0116. **(D)** Quantification from **(B)**. *t*-test, *p-value = 0.0435. Data were pooled from three independent experiments analyzing 50 cells per condition and experiment.

In this experiment, we determined if cultured CEC isolated from aged mice exhibit enhanced levels of G4s. CEC from young and aged mice were stained with antibodies against G4s (BG4). We found that the BG4-positive puncta localized inside and outside the nuclei of aged CEC (Figures 2A,C). Anti-BG4 can recognize both DNA-G4 and RNA-G4. Thus, to evaluate predominantly RNA-G4, we incubated the fixed cells with DNaseI to deplete DNA targets (1 mg/ml, 2 h, 37°C). We found that aged CEC still had higher levels of BG4, mostly located outside of the nuclei, compared with young CEC (Figures 2B,D). These results confirm that enhanced levels of DNA and RNA G4s are features of aging in cultured CEC.

### G4 Stabilization Induces Senescence in Cultured CEC

Next, we tested the hypothesis that G4 stabilization induces senescence in CEC. We can stabilize G4s in cultured cells by using small G4-stabilizing compounds, such as PDS that intercalate between guanines aligned in the G4 planar tetrads of a DNA or RNA sequence.

**FIGURE 3:**
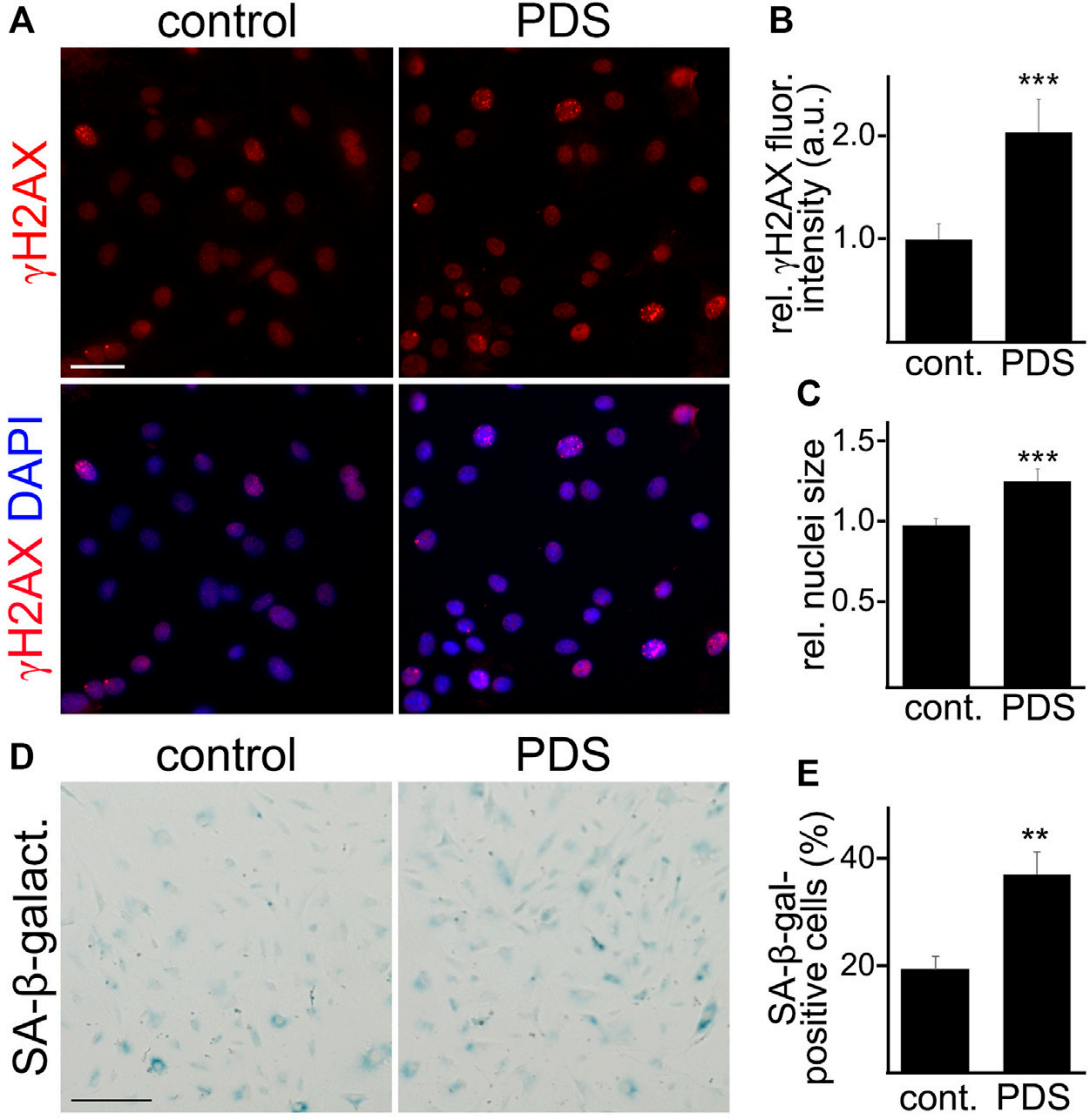
A G4-stabilizing compound exacerbated senescence-associated phenotypes in cultured CEC. **(A)** CEC isolated from aged (18–20 m/o) female mice and cultured were treated with pyridostatin (0.5 µM), or a vehicle (water), for 4 days. CEC were fixed and stained with antibodies against γH2AX and the nuclear Hoechst dye (DAPI channel). Scale bar, 50 µm **(B)** Quantification of the fluorescence intensity of γH2AX from **(A)**. *t*-test, ***p-value = 0.0003. Data were pooled from three independent experiments analyzing 50 cells per condition and experiment. **(C)** Quantification of the nuclei size. *t*-test, ***p-value = 0.0001. Data were pooled from three independent experiments analyzing 50 cells per condition and experiment. **(D)** CEC isolated from aged (18–20 m/o) female mice and cultured were treated with pyridostatin (0.5 µM), or a vehicle (water), for 4 days. CEC were fixed and stained with the SA-β-galactosidase staining kit. Scale bar, 50 µm. **(E)** Quantification of the percentage of SA-β-galactosidase-positive cells. *t*-test, **p-value = 0.0015. Data were pooled from three independent experiments analyzing 50 cells **(A**–**C)** or 20 microscopic fields **(D**,**E)** per condition and experiment.

Given that cultured cells can become senescent days after a genotoxic stimulus (Kirkland and Tchkonia 2017), we treated CEC with a long-term exposure time of PDS at a low concentration. Cultured primary CEC isolated from aged mice were treated either with a low concentration of PDS (0.5 µM), or a vehicle, for 4 days. Cells were fixed and stained with antibodies against γH2AX, and we measured γH2AX fluorescence intensity. CEC treated with PDS showed higher γH2AX fluorescence intensity compared with control cells (Figures 3A,B). We also observed that CEC treated with PDS showed a significant increase in their nuclear size, which is also a feature of cell senescence (Rey et al., 2017; Bang et al., 2021) (Figure 3C). Another cohort of cultured primary CEC was treated either with PDS, or with a vehicle, and stained for SA-β-galactosidase. We found that a higher percentage of PDS-treated CEC was positive for SA-β-galactosidase staining compared with the control cells (Figures 3D,E). Thus, G4 stabilization exacerbated senescence in cultured CEC isolated from aged mice.

### The Cerebral Vasculature and Cultured Primary CEC Isolated From Aged Mice Show Increased Levels of CCN1

**FIGURE 4:**
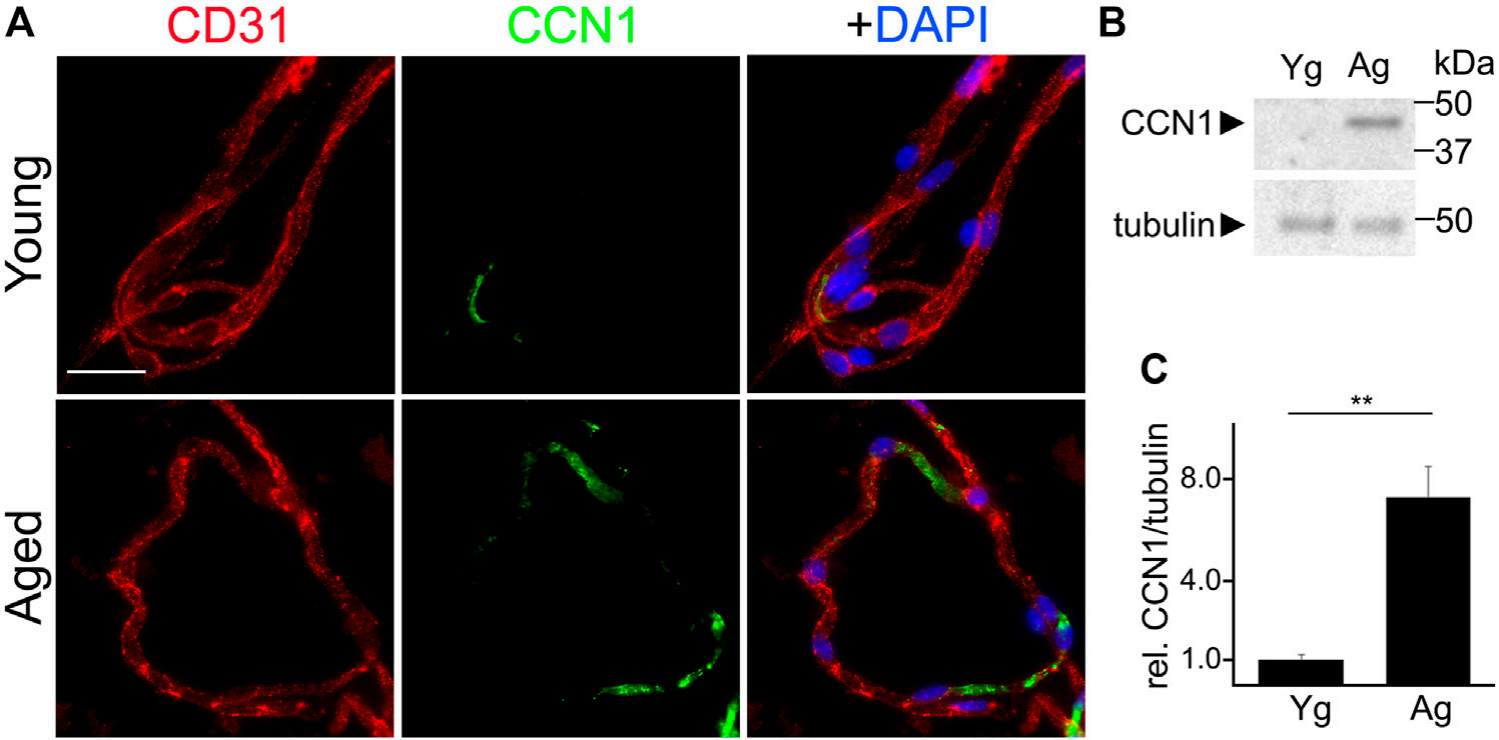
CCN1 was upregulated in the microvessels isolated from the brains of aged mice and in cultured CEC isolated from aged mice compared with young mice. **(A)** Representative image of microvessels isolated from the brains of young (3–4 m/o) and aged (18–20 m/o) female mice, and stained with antibodies against the endothelial marker CD31, with antibodies against CCN1, and with the nuclear Hoechst dye (DAPI channel). Scale bar, 25 µm. **(B)** CEC were isolated from young (3-4- m/o) and aged (18–20 m/o) female mice, cultured and maintained until 100% confluence. Cells were collected and lysed and processed for gel electrophoresis and analyzed for Western blotting. **(C)** Band intensities of CCN1 relative to tubulin. *t*-test, ** *p*-value < 0.0019. Data were obtained from four mice per age.

Enhanced levels of CCN1 have been associated with senescence in non-endothelial cell types (Jun and Lau 2010; Simmnacher et al., 2020). Thus, we hypothesized that CCN1 was upregulated in the cerebral vasculature of aged mice compared with young brains. We isolated cerebral microvessels from the brains of young and aged mice and stained with antibodies against an endothelial marker (CD31), and CCN1, and labeled nuclei with the Hoechst dye. We found that the fluorescence intensity of CCN1 was enhanced in the cerebral microvessels from aged mice compared with young mice (Figure 4A). Similarly, we determined whether cultured primary CEC also recapitulate the enhancement of CCN1 levels with aging as we observed in microvessels from aged mice. Thus, we generated primary CEC from young and aged mice and measured CCN1 levels by Western blotting. The level of CCN1 was significantly increased in CEC from aged mice compared with young mice (Figures 4B,C).

Our data indicate that microvessels and CEC isolated from the brains of aged mice show enhanced SA phenotype compared with those from young mice, and that CCN1 was upregulated in the cerebrovasculature and in cultured primary CEC with aging. These findings suggest that senescence in the cerebral vasculature is associated with enhanced levels of CCN1 in aged mice. However, whether increased CCN1 is a causative factor in promoting senescence in primary CEC remained to be established.

### CCN1 Enhances Senescence Phenotype in Cultured CEC From Aged Mice

**FIGURE 5:**
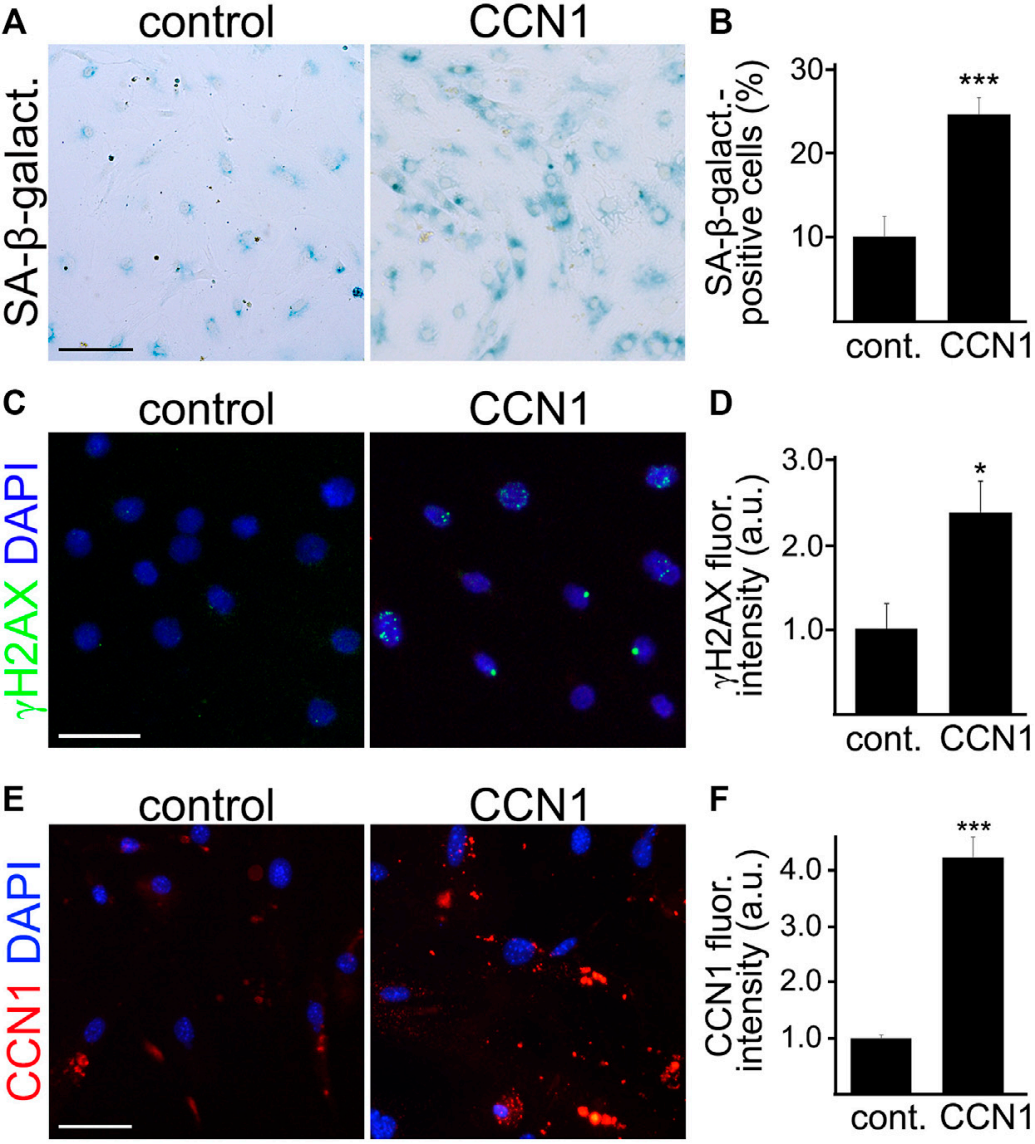
CCN1 induced senescence-associated phenotypes in cultured CEC. CEC were isolated from aged (18–20 m/o) female mice and cultured. Cultured CEC were stained with SA-β-galactosidase staining kit **(A)**, or with antibodies against the double-strand DNA damage marker γH2AX **(C)**, or with antibodies against CCN1 **(E)**, and with the nuclear Hoechst dye **(C**,**E)**. The percentage of SA-β-galactosidase-positive cells **(B)**, the fluorescence intensity of γH2AX **(D)**, and the fluorescence intensity of CCN1 **(F)** were quantified. Scale bar **(A)**, 100 μm; scale bar **(C)**, 50 μm; scale bar **(E)**, 50 μm. *t*-test, ***p-value **(B)** = 0.0001, *n* = 4; *p-value **(D)** = 0.0164, ***p-value **(F)** = 0.0001. Data were pooled from three independent experiments analyzing 20 microscopic fields **(A**,**B**,**E**,**F)** or 50 cells **(C**,**D)** per condition and experiment.

CCN1 induces senescence in cultured human fibroblasts (Jun and Lau 2010), and *Ccn1* is the most upregulated gene in cultured human fetal astrocytes treated with H_2_O_2_, which is commonly used to induce senescence (Simmnacher et al., 2020). However, *Ccn1* is not significantly upregulated in reactive astrocytes (Simmnacher et al., 2020). This highlights the specific relevance of CCN1 to cell senescence. We propose that increased CCN1 promotes senescence in CEC. To test, CEC were isolated from the brains of aged mice and cultured. Cultured CEC were then treated either with recombinant CCN1 (2.5 µM) or vehicle for 4 days and then stained for SA-β-galactosidase. We observed a higher percentage of SA-β-galactosidase positive cells in the CCN1 treated group (Figures 5A,B). Similarly, we observed that CEC treated with CCN1 had significantly higher fluorescence intensities of γH2AX (Figures 5C,D). This indicates that CCN1 can enhance the SA phenotype in aged CEC. Moreover, cultured CEC treated with CCN1 or a vehicle for 4 days were also stained with antibodies against CCN1. We observed that CCN1-treated cells had 4-fold increase of CCN1 levels compared with control cells (Figures 5E,F), suggesting that CCN1 could promote the synthesis of CCN1 in a positive feedback mechanism. This effect may be relevant to the propagation of SA-phenotype to other cells.

### CCN1 Levels Are Upregulated by G4 Stabilization in Cultured CEC

*Ccn1* is transcriptionally activated immediately after exposure to multiple stimuli, such as growth factors (O'Brien et al., 1990; Brunner et al., 1991; Cui et al., 2011), hormones (Hilfiker et al., 2002; Hilfiker-Kleiner et al., 2004), different stressors (including hypoxia (Kunz et al., 2003), UV light (Quan et al., 2006), and mechanical stretch (Chaqour and Goppelt-Struebe 2006; Kivela et al., 2007)), and microorganisms (Kim et al., 2004; Kurozumi et al., 2008; Wiedmaier et al., 2008). However, it is unknown how physiological aging can upregulate CCN1.

A study found that the expression of *CCN1* in human cancer cells could be upregulated *via* the stabilization of G4s in the 3′-UTR of the human *CCN1* mRNA near the stop codon (Sanders et al., 2013). DNA and RNA with guanine sequences intercalated between other bases can fold into G4 structures. Increased G4s have been associated with aging (Lejault et al., 2020; Moruno-Manchon et al., 2020; Teng et al., 2020). Whether CCN1 can be upregulated by G4 stabilization in cultured mouse CEC has not been demonstrated. Thus, we tested the hypothesis that the 3′-UTR of the mouse *Ccn1* mRNA also folds into a G4.

**FIGURE 6:**
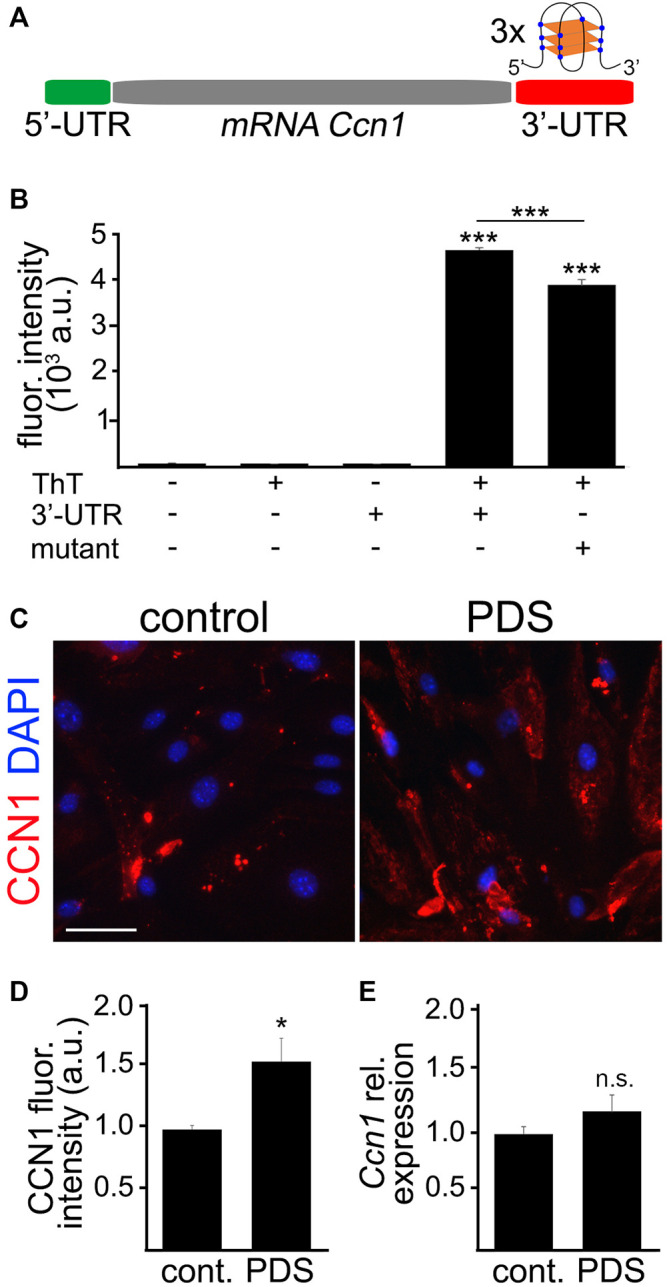
The 3′-UTR *Ccn1* mRNA folded into G4s. **(A)** Scheme of the mRNA *Ccn1* where the 5′-UTR (green) does not contain any potential G4 motif, and the 3′-UTR (red) contains three potential G4 motifs. In the representation of a RNA G4 structure, blue dots indicate Gs and orange rectangles indicate planar rearrangements between Gs. **(B)** The RNA sequence containing the potential G4 in the 3′-UTR *Ccn1* near the stop codon (3′-UTR) or the RNA sequence containing the 3′-UTR *Ccn1* with a single mutation (mutant), as a negative control, were incubated at 90°C for 2 m, and cooled down at room temperature for 2 h. Then, the annealed RNA 3′-UTR and mutant sequences were mixed with a Thioflavin T (ThT) solution, and the fluorescence emission was measured. As controls we used the buffer alone, a ThT solution alone, and the 3′-UTR sequence alone. One-way ANOVA, ***p-value < 0.0001. Data were collected from four independent experiments. **(C)** CEC isolated from aged (18–20 m/o) female mice and cultured were treated with pyridostatin (0.5 µM), or a vehicle (water), for 4 days. CEC were fixed and stained with antibodies against CCN1 and with the nuclear Hoechst dye (DAPI channel). Scale bar, 50 µm. **(D)** Quantification of the fluorescence intensity of CCN1 from **(C)**. *t*-test, *p-value = 0.0103. Data were pooled from three independent experiments analyzing 20 microscopic fields per condition and experiment. **(E)** CEC isolated from aged (18–20 m/o) female mice and cultured were treated with pyridostatin (0.5 µM), or a vehicle, for 4 days. RNA was isolated and qPCR was performed for the relative expression of *Ccn1*. We used *Gapdh* as a housekeeping gene. *t*-test, n.s., non-significant, *p*-value = 0.2424. Data were pooled from four independent experiments.

Using the bioinformatics tool G4Hunter, we found that the mouse *Ccn1* mRNA has three putative G4 motifs in the 3′-UTR and none in the 5′-UTR (Figure 6A). Two of these putative G4s in the 3′-UTR in the mouse *Ccn1* mRNA have a high G4Hunter score (1.148), suggesting that these sequences have the ability to fold into G4s. The sequence located 51 nucleotides after the stop codon (3′-UTR *Ccn1* –G1) shares 100% homology with the G4 motif found in the 3′-UTR in the *CCN1* mRNA of humans. To confirm that the G4 sequence in the 3′-UTR of the *Ccn1* mRNA of mice can indeed fold into a G4, we measured the fluorescence intensity of Thioflavin T (ThT) in combination with an oligo containing the 3′-UTR in *Ccn1* mRNA, or with an oligo with a single modification in a nucleotide from the original 3′-UTR *Ccn1* sequence, which we used as a negative control as it is not predicted to fold into a G4. While ThT can bind to DNA G4 structures (Zhang et al., 2018), it can also bind to other non-G4 DNA forms (Xu et al., 2016). However, when ThT interacts with RNA G4 it causes an enhancement in the fluorescence intensity of the formed complex by several hundred-fold (Renaud de la Faverie et al., 2014; Xu et al., 2016). This increase in fluorescence intensity of ThT does not occur when ThT is incubated with other RNA forms (Xu et al., 2016). Thus, measuring the fluorescence intensity of ThT in combination with a putative RNA G4 sequence is a quick and feasible method to quantify the ability of an oligo to fold into a G4. We observed that the fluorescence intensity of ThT was enhanced by several thousand times when incubated with the 3′-UTR *Ccn1* oligo, compared with the fluorescence of ThT alone or with the fluorescence of the oligonucleotide alone (Figure 6B). We also observed that modifying one single nucleotide in this sequence resulted in a significant reduction of fluorescence intensity. This finding suggests the potential for the 3′-UTR of *Ccn1* RNA to fold into a G4 structure.

Opposite to the G4 stabilization in 5′-UTR motifs, the formation of G4s in 3′-UTR has been associated with enhanced translation (Pelletier and Sonenberg 1985; Koromilas et al., 1992). Given that the 5′-UTR in *Ccn1* mRNA does not contain any putative G4, we hypothesized that the presence of putative G4s in the 3′-UTR in *Ccn1* mRNA can promote the upregulation of CCN1 in cultured CEC. By using the G4-stabilizing compound PDS we can stabilize G4s in cultured cells. Using this approach, we aimed to determine whether CCN1 is upregulated by G4 stabilization in CEC. Thus, cultured primary CEC isolated from aged mice were treated either with PDS (0.5 µM), or with a vehicle, for 4 days. Cells were stained with antibodies against CCN1 and with the nuclear Hoechst dye. We observed that the CCN1 levels were significantly increased in CEC treated with PDS compared with vehicle-treated cells (Figures 6C,D). To determine if PDS affects the expression of *Ccn1*, CEC were incubated with PDS as mentioned and their RNA was collected for qPCR analyses. We found that the relative expression of *Ccn1* in CEC treated with PDS was not significantly different compared with control cells (Figure 6E). This suggests that G4 stabilization regulates CCN1 more importantly at the translational levels than at the transcriptional levels.

## Discussion

Our study indicates that enhanced stabilization of G4s was associated with enhanced SA phenotype in CEC isolated from aged mice. We found that the extracellular component CCN1 was upregulated in the brain microvasculature of aged mice and in cultured CEC from aged mice, compared with young mice. Furthermore, CCN1 was upregulated by G4 stabilization in cultured CEC, likely at the translational level. Thus, our data suggest that the stabilization of RNA-G4s in aging could be an underlying mechanism of cerebrovascular senescence.

Around 10% of CEC have been found to undergo senescence in the brain of aged mice (28-m/o) (Kiss et al., 2020). Senescence of CEC can contribute to cerebrovasculature impairment in a multifaceted manner: dysregulating cerebral blood flow, increasing BBB permeability and changing the cerebrovasculature architecture (Del Valle et al., 2009; Yang et al., 2017; Graves and Baker 2020; Schumacher et al., 2021). Senescence in the brain has been associated with cerebral hypoperfusion (Pincon et al., 2019) and dementia (Zhang et al., 2013; Wang et al., 2018). Alarmingly, the number of individuals affected by dementia, mainly in people after age 65 (Seetlani et al., 2016), is expected to increase to 70 million by 2030 (Wolters and Ikram 2019). Thus, with the increasing number of people living to very advanced ages, the significance of our study is further strengthened. The possibility of modulating the synthesis of senescence-inducing factors, such as CCN1, could potentially prevent or mitigate senescence in the brain vasculature.

The role of CCN1 in senescence has been documented in studies using human fibroblasts (Jun and Lau 2010). CCN1 binds to integrin α_6_β_1_ and heparin sulfate proteoglycans and activates the DNA damage response factors ATM, CHK1, CHK2 and p53. In a reactive oxygen species-dependent manner, the signaling pathways p38 MAPK and ERK are also stimulated by CCN1, and this triggers the p16^INK4a^/pRb pathway, which promotes cellular senescence. CCN1 also induces the expression of matrix metalloproteinases that are part of the SASP. Cells that bind to extracellular CCN1 exhibit higher ROS levels compared with cells adhered to other extracellular matrix components, such as collagen, fibronectin, or laminin. Similar to CCN1, laminin binds to α_6_β_1_ and heparin sulfate proteoglycans; however, it does not induce senescence, like CCN1 does (Jun and Lau 2010). This suggests that senescence induction is a particular feature of CCN1 among other extracellular matrix components, and that CCN1 may act *via* other receptors in addition to α_6_β_1_ and heparin sulfate proteoglycans. Elucidating the CCN1-associated signaling pathways could help to develop therapeutic strategies to mitigate endothelial senescence.

Over 13,000 loci form RNA-G4 within the human transcriptome *in vitro*. Around 3,700 potential RNA-G4s were found in 2,500 genes (Kwok et al., 2016), mostly located in the 5′- and 3′-UTRs of mRNA (Song et al., 2016; Fay et al., 2017). High number of RNA-G4s in the 5′-UTRs interrupt mRNA translation (Pelletier and Sonenberg 1985; Koromilas et al., 1992). However, RNA-G4s in 3′-UTRs can positively regulate translation, where the polyA tail binds to elongation factors, which results in mRNA circulation and enhanced initiation rate (Sonenberg and Hinnebusch 2009). We found that the 5′-UTR in *Ccn1* mRNA does not contain G-rich motifs with the potential ability to fold into G4s. However, its 3′-UTR has three putative G4s and we observed that the closest sequence to the stop codon can fold into RNA-G4 using ThT. This may explain why CCN1 levels were upregulated in cultured CEC treated with the G4-stabilizing molecule PDS. Strikingly, a study found that the 3′-UTR plays a repressive role in the human *CCN1* expression (Nakagawa et al., 2013). The authors found that the proximal half of the 3′-UTR, mainly between the downstream 50 and 160 bases, negatively interferes with the post-transcriptional regulation of *CCN1*. However, the region with the first 160 bases contains the G4-forming sequence that folds into RNA-G4 (Sanders et al., 2013). The experiments in the Nakagawa study were performed under G4 non-inducing conditions. This suggests that the proximal half of the 3′-UTR may repress *CCN1* expression under normal conditions; however, with G4 stabilizers, the 3′-UTR may fold into a G4, and the role of G4s in the 3′-UTR on the post-transcriptional activity in *CCN1* is inverted and thus could promote CCN1 upregulation. G4 ligands with high affinity to RNA-G4 prevent cell proliferation by promoting cell cycle exit (Goldberg et al., 2020), suggesting that the stabilization of RNA-G4s causes cell senescence. Further studies are needed to determine if modifications of nucleotides in the RNA-G4 forming sequence in the 3′-UTR *Ccn1* mRNA could prevent CCN1 upregulation under conditions where G4 stabilization occurs, such as aging.

The role of the G4 stabilization on cell senescence has been investigated in the cancer field. Small G4-stabilizing compounds induce senescence in cancer cells (Riou et al., 2002; Huang et al., 2012), which makes these compounds potential candidates for anticancer therapy. It is generally accepted that a common mechanism of these G4-stabilizers is acceleration of telomere shortening, which is an important feature of aging. However, as G4 stabilization regulates multiple cellular processes (transcription (Armas et al., 2017; Moruno-Manchon et al., 2020), translation (Schaeffer et al., 2001; Khateb et al., 2007), RNA splicing (Zhang et al., 2019), mRNA subcellular localization (Subramanian et al., 2011), and DNA integrity (Moruno-Manchon et al., 2017; Techer et al., 2017)), mechanisms besides telomere shortening may also be involved in cell senescence. For example, DNA damage is the primary cause of senescence and aging (Schumacher et al., 2021). Relevant to the brain, we previously demonstrated that the stabilization of G4s induces DNA damage in cultured primary neurons (Moruno-Manchon et al., 2017; Tabor et al., 2021), and in cultured primary astrocytes and microglia (Tabor et al., 2021). Subventricular zone neural stem and progenitor cells treated with PDS also showed enhanced DNA damage (Goldberg et al., 2020). Autophagy is another process that has been implicated in cell senescence (Rajendran et al., 2019). Autophagy is a self-digesting mechanism that maintains cellular functions and survival. Stimulating autophagy can prevent senescence in vascular smooth muscle cells (Li et al., 2014; Luo et al., 2017). We demonstrated that inducing G4 stabilization inhibited autophagy in cultured neurons (Moruno-Manchon et al., 2020) and astrocytes (Lejault et al., 2020). Furthermore, we demonstrated that aged mice treated with PDS showed enhanced SA phenotype in their brains, such as increased lipofuscin accumulation and DNA damage. These mice also exhibited enhanced memory deficits compared with control aged mice (Moruno-Manchon et al., 2020). Thus, these studies highlight the influence of G4 stability in cell senescence in the brain and cognitive function.

While overall protein synthesis is decreased in senescent cells (Makrides 1983), the levels of mRNAs encoding SASP factors are enhanced with senescence (Coppe et al., 2008). We show evidence that stabilizing G4s can upregulate the senescence-inducing component CCN1 in cultured endothelial cells. Other labs have demonstrated that upregulating CCN members promote senescence and SASP in other cell types (Jun and Lau 2017; Feng et al., 2020), and that downregulating CCN1 reduced the expression of SASP factors (Feng et al., 2020). From this evidence, we hypothesize that other SASP could be regulated by G4s. Indeed, the expression of the SASP component vascular endothelial growth factor (VEGF) is downregulated by G4 stabilization. The Hurley lab was the first to demonstrate that the promoter region of VEGF naturally folds into G4s (Sun et al., 2005; Guo et al., 2008; Sun et al., 2008). Since then, investigators have been developing aptamers to target G4-forming sequences in VEGF promoter as a therapeutic application in cancer. Aptamers are short DNA or RNA sequences that specifically recognize molecules with high affinity. Anti-VEGF DNA-based aptamers forming G4s have been synthesized to visualize and target VEGF (Riccardi et al., 2021). The use of aptamers shows less toxicity and reduced immunogenicity over antibodies, and they are easy to produce. Thus, the development of G4 motif-based aptamers is feasible and has an important therapeutic potential (Duchler 2012) including for preventing or possibly reversing senescence. For example, aptamers have been used in animal models to rejuvenate bone tissue (Aldahmash 2016). We therefore propose that an important strategy could involve identifying G4 motifs is SASP components and that developing G4 motif-based molecules to target these senescence-inducing factors could reverse or prevent senescence in the cerebral endothelium.

A limitation in our study is that our experiments were performed using CEC isolated from only female mice. Studying the effects of CCN1 on senescence in aged female mice is relevant to vascular aging in women. The arteries of postmenopausal women are stiffer than the arteries of age-matched men (Merz and Cheng 2016). Arterial stiffness is associated with increased blood pressure and it is a risk factor for cardiovascular events (Bonarjee 2018). Elderly women have higher rates of hypertension than men (Merz and Cheng 2016). Physiological levels of CCN1 are required for optimal vascular stiffness, as CCN1 loss leads to alterations of vessel structure and integrity (Lee et al., 2019). However, high levels of CCN1 have been associated with pathological vascular stiffness (Reid et al., 2017; Chaqour 2020). Whether sex differences exist in the expression and translation of *Ccn1*, the contributions of gonadal hormones and sex chromosomes (XX vs XY), and their implication in the cerebrovasculature is being actively pursued in our lab.

In conclusion, our study supports a model in which the stabilization of G4s in aging contributes to brain vasculature senescence by regulating the levels of SASP factors such as CCN1. Therefore, developing G4 motif-based molecules to target these senescence-inducing factors should be explored as an approach to reverse or mitigate senescence in the cerebrovasculature.

## Data Availability

The RNAseq data presented in this study can be found in online repositories. The names of the repository/repositories and accession number(s) can be found below: https://www.ncbi.nlm.nih.gov/bioproject/785238.

## References

[B1] Agarwal T. Roy S. Kumar S. Chakraborty T. K. Maiti S. (2014). In the Sense of Transcription Regulation by G-Quadruplexes: Asymmetric Effects in Sense and Antisense Strands. Biochemistry 53 (23), 3711–3718. 10.1021/bi401451q 24850370

[B2] Aldahmash A. (2016). Skeletal Stem Cells and Their Contribution to Skeletal Fragility: Senescence and Rejuvenation. Biogerontology 17 (2), 297–304. 10.1007/s10522-015-9623-7 26510555 PMC4819465

[B3] Armas P. David A. Calcaterra N. B. (2017). Transcriptional Control by G-Quadruplexes: In Vivo Roles and Perspectives for Specific Intervention. Transcription 8 (1), 21–25. 10.1080/21541264.2016.1243505 27696937 PMC5279714

[B4] Bang M. Gonzales E. L. Shin C. Y. Kwon K. J. (2021). Late Passage Cultivation Induces Aged Astrocyte Phenotypes in Rat Primary Cultured Cells. Biomolecules Ther. 29 (2), 144–153. 10.4062/biomolther.2020.175 PMC7921865 33262320

[B5] Bedrat A. Lacroix L. Mergny J.-L. (2016). Re-evaluation of G-Quadruplex Propensity with G4Hunter. Nucleic Acids Res. 44 (4), 1746–1759. 10.1093/nar/gkw006 26792894 PMC4770238

[B6] Bonarjee V. V. S. (2018). Arterial Stiffness: A Prognostic Marker in Coronary Heart Disease. Available Methods and Clinical Application. Front. Cardiovasc. Med. 5, 64. 10.3389/fcvm.2018.00064 29951487 PMC6008540

[B7] Brunner A. Chinn J. Neubauer M. Purchio A. F. (1991). Identification of a Gene Family Regulated by Transforming Growth Factor-β. DNA Cel Biol. 10 (4), 293–300. 10.1089/dna.1991.10.293 2029337

[B8] Castillo Bosch P. Segura‐Bayona S. Koole W. Heteren J. T. Dewar J. M. Tijsterman M. (2014). FANCJ Promotes DNA Synthesis Through G‐quadruplex Structures. EMBO J. 33 (21), 2521–2533. 10.15252/embj.201488663 25193968 PMC4282361

[B9] Chaqour B. (2020). Caught Between a "Rho" and a Hard Place: Are CCN1/CYR61 and CCN2/CTGF the Arbiters of Microvascular Stiffness? J. Cel Commun. Signal. 14 (1), 21–29. 10.1007/s12079-019-00529-3 PMC7176800 31376071

[B10] Chaqour B. Goppelt-Struebe M. (2006). Mechanical Regulation of the Cyr61/CCN1 and CTGF/CCN2 Proteins. FEBS J. 273 (16), 3639–3649. 10.1111/j.1742-4658.2006.05360.x 16856934

[B11] Chen Y. Liu L. (2012). Modern Methods for Delivery of Drugs Across the Blood-Brain Barrier. Adv. Drug Deliv. Rev. 64 (7), 640–665. 10.1016/j.addr.2011.11.010 22154620

[B12] Coppé J. P. Patil C. K. Rodier F. Sun Y. Muñoz D. P. Goldstein J. (2008). Senescence-associated Secretory Phenotypes Reveal Cell-Nonautonomous Functions of Oncogenic RAS and the P53 Tumor Suppressor. Plos Biol. 6 (12), 2853–2868. 10.1371/journal.pbio.0060301 19053174 PMC2592359

[B13] Cui T. X. Lin G. LaPensee C. R. Calinescu A.-A. Rathore M. Streeter C. (2011). C/EBPβ Mediates Growth Hormone-Regulated Expression of Multiple Target Genes. Mol. Endocrinol. 25 (4), 681–693. 10.1210/me.2010-0232 21292824 PMC3063086

[B14] Dimri G. P. Lee X. Basile G. Acosta M. Scott G. Roskelley C. (1995). A Biomarker that Identifies Senescent Human Cells in Culture and in Aging Skin In Vivo. Proc. Natl. Acad. Sci. 92 (20), 9363–9367. 10.1073/pnas.92.20.9363 7568133 PMC40985

[B15] Düchler M. (2012). G-quadruplexes: Targets and Tools in Anticancer Drug Design. J. Drug Target. 20 (5), 389–400. 10.3109/1061186x.2012.669384 22424091

[B16] Fay M. M. Lyons S. M. Ivanov P. (2017). RNA G-Quadruplexes in Biology: Principles and Molecular Mechanisms. J. Mol. Biol. 429 (14), 2127–2147. 10.1016/j.jmb.2017.05.017 28554731 PMC5603239

[B17] Feng M. Peng H. Yao R. Zhang Z. Mao G. Yu H. (2020). Inhibition of Cellular Communication Network Factor 1 (CCN1)-Driven Senescence Slows Down Cartilage Inflammaging and Osteoarthritis. Bone 139, 115522. 10.1016/j.bone.2020.115522 32622876

[B18] Goldberg D. C. Fones L. Vivinetto A. L. Caufield J. T. Ratan R. R. Cave J. W. (2020). Manipulating Adult Neural Stem and Progenitor Cells with G-Quadruplex Ligands. ACS Chem. Neurosci. 11 (10), 1504–1518. 10.1021/acschemneuro.0c00194 32315155

[B19] Graves S. I. Baker D. J. (2020). Implicating Endothelial Cell Senescence to Dysfunction in the Ageing and Diseased Brain. Basic Clin. Pharmacol. Toxicol. 127 (2), 102–110. 10.1111/bcpt.13403 32162446 PMC7384943

[B20] Guo K. Gokhale V. Hurley L. H. Sun D. (2008). Intramolecularly Folded G-Quadruplex and I-Motif Structures in the Proximal Promoter of the Vascular Endothelial Growth Factor Gene. Nucleic Acids Res. 36 (14), 4598–4608. 10.1093/nar/gkn380 18614607 PMC2504309

[B21] Hilfiker A. Hilfiker-Kleiner D. Fuchs M. Kaminski K. Lichtenberg A. Rothkötter H.-J. (2002). Expression of CYR61, an Angiogenic Immediate Early Gene, in Arteriosclerosis and its Regulation by Angiotensin II. Circulation 106 (2), 254–260. 10.1161/01.cir.0000021426.87274.62 12105167

[B22] Hilfiker-Kleiner D. Kaminski K. Kaminska A. Fuchs M. Klein G. Podewski E. (2004). Regulation of Proangiogenic Factor CCN1 in Cardiac Muscle. Circulation 109 (18), 2227–2233. 10.1161/01.cir.0000127952.90508.9d 15117851

[B23] Huang F.-C. Chang C.-C. Wang J.-M. Chang T.-C. Lin J.-J. (2012). Induction of Senescence in Cancer Cells by the G-Quadruplex Stabilizer, BMVC4, Is Independent of its Telomerase Inhibitory Activity. Br. J. Pharmacol. 167 (2), 393–406. 10.1111/j.1476-5381.2012.01997.x 22509942 PMC3481046

[B24] Jia G. Aroor A. R. Jia C. Sowers J. R. (2019). Endothelial Cell Senescence in Aging-Related Vascular Dysfunction. Biochim. Biophys. Acta (Bba) - Mol. Basis Dis. 1865 (7), 1802–1809. 10.1016/j.bbadis.2018.08.008 31109450

[B25] Jun J.-I. Lau L. F. (2017). CCN2 Induces Cellular Senescence in Fibroblasts. J. Cel Commun. Signal. 11 (1), 15–23. 10.1007/s12079-016-0359-1 PMC5362572 27752926

[B26] Jun J.-I. Lau L. F. (2010). The Matricellular Protein CCN1 Induces Fibroblast Senescence and Restricts Fibrosis in Cutaneous Wound Healing. Nat. Cel Biol 12 (7), 676–685. 10.1038/ncb2070 PMC2919364 20526329

[B27] Khateb S. Weisman-Shomer P. Hershco-Shani I. Ludwig A. L. Fry M. (2007). The Tetraplex (CGG)n Destabilizing Proteins hnRNP A2 and CBF-A Enhance the In Vivo Translation of Fragile X Premutation mRNA. Nucleic Acids Res. 35 (17), 5775–5788. 10.1093/nar/gkm636 17716999 PMC2034458

[B28] Kim S.-M. Park J.-H. Chung S.-K. Kim J.-Y. Hwang H.-Y. Chung K.-C. (2004). Coxsackievirus B3 Infection Induces Cyr61 Activation via JNK to Mediate Cell Death. J. Virol. 78 (24), 13479–13488. 10.1128/jvi.78.24.13479-13488.2004 15564459 PMC533934

[B29] Kirkland J. L. Tchkonia T. (2017). Cellular Senescence: A Translational Perspective. EBioMedicine 21, 21–28. 10.1016/j.ebiom.2017.04.013 28416161 PMC5514381

[B30] Kiss T. Nyúl-Tóth Á. Balasubramanian P. Tarantini S. Ahire C. DelFavero J. (2020). Single-cell RNA Sequencing Identifies Senescent Cerebromicrovascular Endothelial Cells in the Aged Mouse Brain. Geroscience 42 (2), 429–444. 10.1007/s11357-020-00177-1 32236824 PMC7205992

[B31] Kivelä R. Kyröläinen H. Selänne H. Komi P. V. Kainulainen H. Vihko V. (20071985). A Single Bout of Exercise with High Mechanical Loading Induces the Expression of Cyr61/CCN1 and CTGF/CCN2 in Human Skeletal Muscle. J. Appl. Physiol. 103 (4), 1395–1401. 10.1152/japplphysiol.00531.2007 17673559

[B32] Koromilas A. E. Lazaris-Karatzas A. Sonenberg N. (1992). mRNAs Containing Extensive Secondary Structure in Their 5′ Non-coding Region Translate Efficiently in Cells Overexpressing Initiation Factor eIF-4E. EMBO J. 11 (11), 4153–4158. 10.1002/j.1460-2075.1992.tb05508.x 1396596 PMC556925

[B33] Kruisselbrink E. Guryev V. Brouwer K. Pontier D. B. Cuppen E. Tijsterman M. (2008). Mutagenic Capacity of Endogenous G4 DNA Underlies Genome Instability in FANCJ-Defective *C. elegans* . Curr. Biol. 18 (12), 900–905. 10.1016/j.cub.2008.05.013 18538569

[B34] Kunz M. Moeller S. Koczan D. Lorenz P. Wenger R. H. Glocker M. O. (2003). Mechanisms of Hypoxic Gene Regulation of Angiogenesis Factor Cyr61 in Melanoma Cells. J. Biol. Chem. 278 (46), 45651–45660. 10.1074/jbc.m301373200 12939282

[B35] Kurozumi K. Hardcastle J. Thakur R. Shroll J. Nowicki M. Otsuki A. (2008). Oncolytic HSV-1 Infection of Tumors Induces Angiogenesis and Upregulates CYR61. Mol. Ther. 16 (8), 1382–1391. 10.1038/mt.2008.112 18545226 PMC2659780

[B36] Kwok C. K. Marsico G. Sahakyan A. B. Chambers V. S. Balasubramanian S. (2016). rG4-seq Reveals Widespread Formation of G-Quadruplex Structures in the Human Transcriptome. Nat. Methods 13 (10), 841–844. 10.1038/nmeth.3965 27571552

[B37] Lähteenvuo J. Rosenzweig A. (2012). Effects of Aging on Angiogenesis. Circ. Res. 110 (9), 1252–1264. 10.1161/circresaha.111.246116 22539758 PMC4101916

[B38] Lee S. Ahad A. Luu M. Moon S. Caesar J. Cardoso W. V. (2019). CCN1-Yes-Associated Protein Feedback Loop Regulates Physiological and Pathological Angiogenesis. Mol. Cel Biol 39 (18), e00107-19. 10.1128/MCB.00107-19 PMC6712939 31262999

[B39] Lee Y.-K. Uchida H. Smith H. Ito A. Sanchez T. (2019). The Isolation and Molecular Characterization of Cerebral Microvessels. Nat. Protoc. 14 (11), 3059–3081. 10.1038/s41596-019-0212-0 31586162 PMC11571963

[B40] Lejault P. Moruno-Manchon J. F. Vemu S. M. Honarpisheh P. Zhu L. Kim N. (2020). Regulation of Autophagy by DNA G-Quadruplexes. Autophagy 16 (12), 2252–2259. 10.1080/15548627.2020.1769991 32420812 PMC7751500

[B41] Li X. Xu M. Pitzer A. L. Xia M. Boini K. M. Li P.-L. (2014). Control of Autophagy Maturation by Acid Sphingomyelinase in Mouse Coronary Arterial Smooth Muscle Cells: Protective Role in Atherosclerosis. J. Mol. Med. 92 (5), 473–485. 10.1007/s00109-014-1120-y 24463558 PMC4211081

[B42] Luo Z. Xu W. Ma S. Qiao H. Gao L. Zhang R. (2017). Moderate Autophagy Inhibits Vascular Smooth Muscle Cell Senescence to Stabilize Progressed Atherosclerotic Plaque via the mTORC1/ULK1/ATG13 Signal Pathway. Oxid Med. Cel Longev 2017, 3018190. 10.1155/2017/3018190 PMC5497616 28713484

[B43] Makrides S. C. (1983). Protein Synthesis and Degradation During Aging and Senescence. Biol. Rev. 58 (3), 343–422. 10.1111/j.1469-185x.1983.tb00394.x 6354285

[B44] Merz A. A. Cheng S. (2016). Sex Differences in Cardiovascular Ageing. Heart 102 (11), 825–831. 10.1136/heartjnl-2015-308769 26917537 PMC5993677

[B45] Moreno-Blas D. Gorostieta-Salas E. Pommer-Alba A. Muciño-Hernández G. Gerónimo-Olvera C. Maciel-Barón L. A. (2019). Cortical Neurons Develop a Senescence-like Phenotype Promoted by Dysfunctional Autophagy. Aging 11 (16), 6175–6198. 10.18632/aging.102181 31469660 PMC6738425

[B46] Moruno-Manchon J. F. Lejault P. Wang Y. McCauley B. Honarpisheh P. Morales Scheihing D. A. (2020). Small-molecule G-Quadruplex Stabilizers Reveal a Novel Pathway of Autophagy Regulation in Neurons. Elife 9, e52283. 10.7554/eLife.52283 32043463 PMC7012600

[B47] Moruno-Manchon J. F. Koellhoffer E. C. Gopakumar J. Hambarde S. Kim N. McCullough L. D. (2017). The G-Quadruplex DNA Stabilizing Drug Pyridostatin Promotes DNA Damage and Downregulates Transcription of Brca1 in Neurons. Aging 9 (9), 1957–1970. 10.18632/aging.101282 28904242 PMC5636668

[B48] Nakagawa Y. Minato M. Sumiyoshi K. Maeda A. Hara C. Murase Y. (2013). Regulation of CCN1 via the 3′-untranslated Region. J. Cel Commun. Signal. 7 (3), 207–217. 10.1007/s12079-013-0202-x PMC3709046 23677691

[B49] Noubissi F. K. McBride A. A. Leppert H. G. Millet L. J. Wang X. Davern S. M. (2021). Detection and Quantification of γ-H2AX Using a Dissociation Enhanced Lanthanide Fluorescence Immunoassay. Sci. Rep. 11 (1), 8945. 10.1038/s41598-021-88296-3 33903655 PMC8076281

[B50] O'Brien T. P. Yang G. P. Sanders L. Lau L. F. (1990). Expression of Cyr61, a Growth Factor-Inducible Immediate-Early Gene. Mol. Cel. Biol. 10 (7), 3569–3577. 10.1128/mcb.10.7.3569 PMC360792 2355916

[B51] Oh K. S. Febres-Aldana C. A. Kuritzky N. Ujueta F. Arenas I. A. Sriganeshan V. (2021). Cellular Senescence Evaluated by P16INK4a Immunohistochemistry Is a Prevalent Phenomenon in Advanced Calcific Aortic Valve Disease. Cardiovasc. Pathol. 52, 107318. 10.1016/j.carpath.2021.107318 33450362

[B52] Pelegrí C. Canudas A. M. del Valle J. Casadesus G. Smith M. A. Camins A. (2007). Increased Permeability of Blood-Brain Barrier on the hippocampus of a Murine Model of Senescence. Mech. Ageing Development 128 (9), 522–528. 10.1016/j.mad.2007.07.002 17697702

[B53] Pelletier J. Sonenberg N. (1985). Insertion Mutagenesis to Increase Secondary Structure within the 5′ Noncoding Region of a Eukaryotic mRNA Reduces Translational Efficiency. Cell 40 (3), 515–526. 10.1016/0092-8674(85)90200-4 2982496

[B54] Perrière N. Demeuse P. Garcia E. Regina A. Debray M. Andreux J.-P. (2005). Puromycin-based Purification of Rat Brain Capillary Endothelial Cell Cultures. Effect on the Expression of Blood-Brain Barrier-specific Properties. J. Neurochem. 93 (2), 279–289. 10.1111/j.1471-4159.2004.03020.x 15816851

[B55] Pinçon A. De Montgolfier O. Akkoyunlu N. Daneault C. Pouliot P. Villeneuve L. (2019). Non-Alcoholic Fatty Liver Disease, and the Underlying Altered Fatty Acid Metabolism, Reveals Brain Hypoperfusion and Contributes to the Cognitive Decline in APP/PS1 Mice. Metabolites 9 (5), 104. 10.3390/metabo9050104 PMC6572466 31130652

[B56] Quan T. He T. Shao Y. Lin L. Kang S. Voorhees J. J. (2006). Elevated Cysteine-Rich 61 Mediates Aberrant Collagen Homeostasis in Chronologically Aged and Photoaged Human Skin. Am. J. Pathol. 169 (2), 482–490. 10.2353/ajpath.2006.060128 16877350 PMC1698795

[B57] Rajendran P. Alzahrani A. M. Hanieh H. N. Kumar S. A. Ben Ammar R. Rengarajan T. (2019). Autophagy and Senescence: A New Insight in Selected Human Diseases. J. Cel Physiol 234 (12), 21485–21492. 10.1002/jcp.28895 31144309

[B58] Reid S. E. Kay E. J. Neilson L. J. Henze A. T. Serneels J. McGhee E. J. (2017). Tumor Matrix Stiffness Promotes Metastatic Cancer Cell Interaction with the Endothelium. EMBO J. 36 (16), 2373–2389. 10.15252/embj.201694912 28694244 PMC5556271

[B59] Renaud de la Faverie A. Guédin A. Bedrat A. Yatsunyk L. A. Mergny J.-L. (2014). Thioflavin T as a Fluorescence Light-Up Probe for G4 Formation. Nucleic Acids Res. 42 (8), e65. 10.1093/nar/gku111 24510097 PMC4005661

[B60] Rey S. Quintavalle C. Burmeister K. Calabrese D. Schlageter M. Quagliata L. (2017). Liver Damage and Senescence Increases in Patients Developing Hepatocellular Carcinoma. J. Gastroenterol. Hepatol. 32 (8), 1480–1486. 10.1111/jgh.13717 28052383

[B61] Riccardi C. Napolitano E. Platella C. Musumeci D. Melone M. A. B. Montesarchio D. (2021). Anti‐VEGF DNA‐based Aptamers in Cancer Therapeutics and Diagnostics. Med. Res. Rev. 41 (1), 464–506. 10.1002/med.21737 33038031

[B62] Riou J. F. Guittat L. Mailliet P. Laoui A. Renou E. Petitgenet O. (2002). Cell Senescence and Telomere Shortening Induced by a New Series of Specific G-Quadruplex DNA Ligands. Proc. Natl. Acad. Sci. 99 (5), 2672–2677. 10.1073/pnas.052698099 11854467 PMC122406

[B63] Salazar G. Cullen A. Huang J. Zhao Y. Serino A. Hilenski L. (2020). SQSTM1/p62 and PPARGC1A/PGC-1alpha at the Interface of Autophagy and Vascular Senescence. Autophagy 16 (6), 1092–1110. 10.1080/15548627.2019.1659612 31441382 PMC7469683

[B64] Sanders P. G. T. Cotterell J. Sharpe J. Isalan M. (2013). Transfecting RNA Quadruplexes Results in Few Transcriptome Perturbations. RNA Biol. 10 (2), 205–210. 10.4161/rna.22781 23235467 PMC3594279

[B65] Schaeffer C. Bardoni B. Mandel J. L. Ehresmann B. Ehresmann C. Moine H. (2001). The Fragile X Mental Retardation Protein Binds Specifically to its mRNA via a Purine Quartet Motif. EMBO J. 20 (17), 4803–4813. 10.1093/emboj/20.17.4803 11532944 PMC125594

[B66] Schumacher B. Pothof J. Vijg J. Hoeijmakers J. H. J. (2021). The Central Role of DNA Damage in the Ageing Process. Nature 592 (7856), 695–703. 10.1038/s41586-021-03307-7 33911272 PMC9844150

[B67] Seetlani N. K. Kumar N. Imran K. Ali A. Shams N. Sheikh T. (2016). Alzheimer and Vascular Dementia in the Elderly Patients. Pak J. Med. Sci. 32 (5), 1286–1290. 10.12669/pjms.325.10792 27882038 PMC5103150

[B68] Siddiqui-Jain A. Grand C. L. Bearss D. J. Hurley L. H. (2002). Direct Evidence for a G-Quadruplex in a Promoter Region and its Targeting with a Small Molecule to Repress C-MYC Transcription. Proc. Natl. Acad. Sci. 99 (18), 11593–11598. 10.1073/pnas.182256799 12195017 PMC129314

[B69] Simmnacher K. Krach F. Schneider Y. Alecu J. E. Mautner L. Klein P. (2020). Unique Signatures of Stress-Induced Senescent Human Astrocytes. Exp. Neurol. 334, 113466. 10.1016/j.expneurol.2020.113466 32949572

[B70] Sonenberg N. Hinnebusch A. G. (2009). Regulation of Translation Initiation in Eukaryotes: Mechanisms and Biological Targets. Cell 136 (4), 731–745. 10.1016/j.cell.2009.01.042 19239892 PMC3610329

[B71] Song H. W. Foreman K. L. Gastfriend B. D. Kuo J. S. Palecek S. P. Shusta E. V. (2020). Transcriptomic Comparison of Human and Mouse Brain Microvessels. Sci. Rep. 10 (1), 12358. 10.1038/s41598-020-69096-7 32704093 PMC7378255

[B72] Song J. Perreault J.-P. Topisirovic I. Richard S. (2016). RNA G-Quadruplexes and Their Potential Regulatory Roles in Translation. Translation 4 (2), e1244031. 10.1080/21690731.2016.1244031 28090421 PMC5173311

[B73] Subramanian M. Rage F. Tabet R. Flatter E. Mandel J. L. Moine H. (2011). G-quadruplex RNA Structure as a Signal for Neurite mRNA Targeting. EMBO Rep. 12 (7), 697–704. 10.1038/embor.2011.76 21566646 PMC3128965

[B74] Sun D. Guo K. Rusche J. J. Hurley L. H. (2005). Facilitation of a Structural Transition in the Polypurine/polypyrimidine Tract within the Proximal Promoter Region of the Human VEGF Gene by the Presence of Potassium and G-Quadruplex-Interactive Agents. Nucleic Acids Res. 33 (18), 6070–6080. 10.1093/nar/gki917 16239639 PMC1266068

[B75] Sun D. Liu W.-J. Guo K. Rusche J. J. Ebbinghaus S. Gokhale V. (2008). The Proximal Promoter Region of the Human Vascular Endothelial Growth Factor Gene Has a G-Quadruplex Structure that Can Be Targeted by G-Quadruplex-Interactive Agents. Mol. Cancer Ther. 7 (4), 880–889. 10.1158/1535-7163.mct-07-2119 18413801 PMC2367258

[B76] Sunderland P. Augustyniak J. Lenart J. Bużańska L. Carlessi L. Delia D. (2020). ATM-deficient Neural Precursors Develop Senescence Phenotype with Disturbances in Autophagy. Mech. Ageing Development 190, 111296. 10.1016/j.mad.2020.111296 32621937

[B77] Tabor N. Ngwa C. Mitteaux J. Meyer M. D. Moruno-Manchon J. F. Zhu L. (2021). Differential Responses of Neurons, Astrocytes, and Microglia to G-Quadruplex Stabilization. Aging 13 (12), 15917–15941. 10.18632/aging.203222 34139671 PMC8266374

[B78] Tchkonia T. Zhu Y. van Deursen J. Campisi J. Kirkland J. L. (2013). Cellular Senescence and the Senescent Secretory Phenotype: Therapeutic Opportunities. J. Clin. Invest. 123 (3), 966–972. 10.1172/jci64098 23454759 PMC3582125

[B79] Técher H. Koundrioukoff S. Nicolas A. Debatisse M. (2017). The Impact of Replication Stress on Replication Dynamics and DNA Damage in Vertebrate Cells. Nat. Rev. Genet. 18 (9), 535–550. 10.1038/nrg.2017.46 28714480

[B80] Teng F.-Y. Wang T.-T. Guo H.-L. Xin B.-G. Sun B. Dou S.-X. (2020). The HRDC Domain Oppositely Modulates the Unwinding Activity of *E. coli* RecQ Helicase on Duplex DNA and G-Quadruplex. J. Biol. Chem. 295 (51), 17646–17658. 10.1074/jbc.ra120.015492 33454004 PMC7762929

[B81] Valle J. Duran‐Vilaregut J. Manich G. Camins A. Pallàs M. Vilaplana J. (2009). Time‐course of Blood-Brain Barrier Disruption in Senescence‐accelerated Mouse Prone 8 (SAMP8) Mice. Int. J. Dev. Neurosci. 27 (1), 47–52. 10.1016/j.ijdevneu.2008.10.002 18992318

[B82] Wang F. Cao Y. Ma L. Pei H. Rausch W. D. Li H. (2018). Dysfunction of Cerebrovascular Endothelial Cells: Prelude to Vascular Dementia. Front. Aging Neurosci. 10, 376. 10.3389/fnagi.2018.00376 30505270 PMC6250852

[B83] Wiedmaier N. Müller S. Köberle M. Manncke B. Krejci J. Autenrieth I. B. (2008). Bacteria Induce CTGF and CYR61 Expression in Epithelial Cells in a Lysophosphatidic Acid Receptor-dependent Manner. Int. J. Med. Microbiol. 298 (3-4), 231–243. 10.1016/j.ijmm.2007.06.001 17765657

[B84] Wolters F. J. Ikram M. A. (2019). Epidemiology of Vascular Dementia. Atvb 39 (8), 1542–1549. 10.1161/atvbaha.119.311908 31294622

[B85] Xu S. Li Q. Xiang J. Yang Q. Sun H. Guan A. (2016). Thioflavin T as an Efficient Fluorescence Sensor for Selective Recognition of RNA G-Quadruplexes. Sci. Rep. 6, 24793. 10.1038/srep24793 27098781 PMC4838840

[B86] Yamazaki Y. Baker D. J. Tachibana M. Liu C.-C. van Deursen J. M. Brott T. G. (2016). Vascular Cell Senescence Contributes to Blood-Brain Barrier Breakdown. Stroke 47 (4), 1068–1077. 10.1161/strokeaha.115.010835 26883501 PMC4811685

[B87] Yang T. Sun Y. Lu Z. Leak R. K. Zhang F. (2017). The Impact of Cerebrovascular Aging on Vascular Cognitive Impairment and Dementia. Ageing Res. Rev. 34, 15–29. 10.1016/j.arr.2016.09.007 27693240 PMC5250548

[B88] Zhang J. Harvey S. E. Cheng C. (2019). A High-Throughput Screen Identifies Small Molecule Modulators of Alternative Splicing by Targeting RNA G-Quadruplexes. Nucleic Acids Res. 47 (7), 3667–3679. 10.1093/nar/gkz036 30698802 PMC6468248

[B89] Zhang S. Sun H. Chen H. Li Q. Guan A. Wang L. (2018). Direct Visualization of Nucleolar G-Quadruplexes in Live Cells by Using a Fluorescent Light-Up Probe. Biochim. Biophys. Acta (Bba) - Gen. Subjects 1862 (5), 1101–1106. 10.1016/j.bbagen.2018.01.022 29410183

[B90] Zhang X. Li G. Guo L. Nie K. Jia Y. Zhao L. (2013). Age-related Alteration in Cerebral Blood Flow and Energy Failure Is Correlated with Cognitive Impairment in the Senescence-Accelerated Prone Mouse Strain 8 (SAMP8). Neurol. Sci. 34 (11), 1917–1924. 10.1007/s10072-013-1407-8 23563860

