# Supplementary material for: G-quadruplexes Stabilization Upregulates CCN1 and Accelerates Aging in Cultured Cerebral Endothelial Cells
Source: Front Aging. 2022 Jan 12;2:797562. doi: 10.3389/fragi.2021.797562 (PMC9261356; doi:10.3389/fragi.2021.797562)
Supplement: Supplementary file 1 [file Table1.docx]

| **Supplemental Table 1. 40% of the 1,000 most upregulated genes in CEC from young male mice were also upregulated in the microvessels of young male mice.** | | | | | | | | | |
| --- | --- | --- | --- | --- | --- | --- | --- | --- | --- |
| *Abcg2* | *Basp1* | *Cox7a2l* | *Etfb* | *Hsp90b1* | *Ncl* | *Psmb1* | *Rpl31* | *Rps8* | *Tmsb4x* |
| *Aco2* | *Bgn* | *Cox7c* | *Fads1* | *Hspa5* | *Ndfip1* | *Psmb4* | *Rpl32* | *Rps9* | *Tpi1* |
| *Actb* | *Bin1* | *Cox8a* | *Fau* | *Hspa8* | *Ndufa4* | *Psmb6* | *Rpl35a* | *Rpsa* | *Tpm1* |
| *Actg1* | *Brk1* | *Cpe* | *Fkbp1a* | *Hspa9* | *Ndufa8* | *Psmb7* | *Rpl36* | *Rrp1* | *Tpm4* |
| *Actn4* | *Bsg* | *Cs* | *Fn1* | *Hspe1* | *Ndufb10* | *Psmc1* | *Rpl36a* | *Rtn4* | *Tpt1* |
| *Actr1a* | *Cald1* | *Csde1* | *Fth1* | *Id1* | *Ndufb11* | *Psmc5* | *Rpl37* | *S100a1* | *Tspan3* |
| *Actr3* | *Calm1* | *Csnk1g2* | *Ftl1* | *Id3* | *Ndufb9* | *Psmd3* | *Rpl37a* | *Scd1* | *Tuba1a* |
| *Add1* | *Calm2* | *Cst3* | *Fus* | *Ifitm3* | *Ndufs2* | *Psmd7* | *Rpl39* | *Scd2* | *Tuba1b* |
| *Aes* | *Calm3* | *Ctbp1* | *Gabarap* | *Igfbp7* | *Ndufs6* | *Ptma* | *Rpl4* | *Sdha* | *Tubb2a* |
| *Ahsa1* | *Calr* | *Ctnna1* | *Gapdh* | *Itgb1* | *Nedd4* | *Ptms* | *Rpl41* | *Sepp1* | *Tubb4b* |
| *Akr1a1* | *Canx* | *Ctnnb1* | *Gdi2* | *Itm2b* | *Nedd8* | *Ptp4a2* | *Rpl5* | *Sepw1* | *Tubb5* |
| *Aldoa* | *Cap1* | *Ctsb* | *Gja1* | *Itm2c* | *Nfe2l1* | *Rab2a* | *Rpl6* | *Serinc1* | *Txn1* |
| *Anapc5* | *Capn2* | *Ctsd* | *Gltp* | *Klc1* | *Nfkbia* | *Rab5c* | *Rpl7* | *Serpine2* | *Uba1* |
| *Anp32b* | *Capns1* | *Ctsl* | *Gltscr2* | *Klf4* | *Ngfrap1* | *Rab7* | *Rpl8* | *Sf3b2* | *Ubb* |
| *Anxa3* | *Capzb* | *Cyb5r3* | *Glud1* | *Lamp1* | *Npm1* | *Rabac1* | *Rpl9* | *Sfr1* | *Ube2d3* |
| *Anxa5* | *Cct4* | *D8Ertd738e* | *Glul* | *Laptm4a* | *Nudc* | *Rac1* | *Rplp1* | *Sh3bgrl3* | *Ubxn1* |
| *Ap2m1* | *Cct5* | *Dbi* | *Gnai2* | *Lars2* | *Nudt4* | *Rad21* | *Rplp2* | *Skp1a* | *Uck2* |
| *Aplp2* | *Cct6a* | *Ddb1* | *Gnb1* | *Ldha* | *P4hb* | *Rap1b* | *Rps10* | *Slc25a3* | *Uqcr10* |
| *Apod* | *Cct8* | *Ddx3x* | *Gnb2* | *Ly6e* | *Pabpc1* | *Rbbp7* | *Rps11* | *Slc25a4* | *Uqcr11* |
| *Apoe* | *Cd151* | *Ddx5* | *Gpi1* | *Maged1* | *Pdha1* | *Rhoa* | *Rps12* | *Slc25a5* | *Uqcrc1* |
| *App* | *Cd63* | *Drap1* | *Gpx1* | *Map4k4* | *Pdia3* | *Rhob* | *Rps13* | *Slc7a5* | *Usmg5* |
| *Arf3* | *Cd81* | *Dynlrb1* | *Gpx4* | *Map7d1* | *Pdia6* | *Rnf10* | *Rps14* | *Smdt1* | *Vcp* |
| *Arl6ip1* | *Cdc42* | *Edf1* | *Gstp1* | *Mapk3* | *Pea15a* | *Rnf187* | *Rps15* | *Snx3* | *Vdac1* |
| *Arl6ip5* | *Cdh5* | *Eef1a1* | *H2afz* | *Mcl1* | *Pebp1* | *Rpl10* | *Rps16* | *Sod1* | *Vdac2* |
| *Arpc1b* | *Cdk16* | *Eef1b2* | *H2-D1* | *Mdh2* | *Pgk1* | *Rpl11* | *Rps17* | *Sparc* | *Vim* |
| *Arpc2* | *Cenpb* | *Eef1g* | *H2-K1* | *Mfge8* | *Pgrmc1* | *Rpl12* | *Rps18* | *Sptan1* | *Wbp5* |
| *Arpc3* | *Cfl1* | *Eef2* | *H3f3b* | *Mgp* | *Pkm* | *Rpl13* | *Rps19* | *Sptbn1* | *Ybx1* |
| *Atf4* | *Chd4* | *Eif1* | *Hdgf* | *Mif* | *Polr2f* | *Rpl13a* | *Rps2* | *Sqstm1* | *Ywhab* |
| *Atp1a1* | *Chmp4b* | *Eif3a* | *Hint1* | *Mlf2* | *Pomp* | *Rpl14* | *Rps21* | *Srp14* | *Ywhae* |
| *Atp1b1* | *Chp1* | *Eif3c* | *Hmgcs1* | *Morf4l2* | *Ppia* | *Rpl17* | *Rps23* | *Stmn1* | *Ywhag* |
| *Atp2a2* | *Cldn11* | *Eif3e* | *Hmgn1* | *Mrfap1* | *Ppp1ca* | *Rpl18* | *Rps25* | *Swi5* | *Ywhah* |
| *Atp5a1* | *Clic4* | *Eif3f* | *Hnrnpa1* | *Msn* | *Ppp2r1a* | *Rpl18a* | *Rps26* | *Tagln2* | *Ywhaq* |
| *Atp5b* | *Clstn1* | *Eif3g* | *Hnrnpa2b1* | *Mt1* | *Prdx2* | *Rpl19* | *Rps27* | *Taldo1* | *Ywhaz* |
| *Atp5c1* | *Clta* | *Eif3h* | *Hnrnpab* | *Mt2* | *Prdx5* | *Rpl21* | *Rps27a* | *Tbca* | *Zwint* |
| *Atp5g3* | *Cnbp* | *Eif4a1* | *Hnrnpk* | *Mtch1* | *Prdx6* | *Rpl22l1* | *Rps29* | *Tcf25* |  |
| *Atp5h* | *Col4a1* | *Eif4b* | *Hnrnpm* | *Mtpn* | *Prelid1* | *Rpl23* | *Rps3* | *Tecr* |  |
| *Atp5j* | *Cops6* | *Eif4g2* | *Hprt* | *Myh9* | *Prkar1a* | *Rpl26* | *Rps3a1* | *Tm4sf1* |  |
| *Atp5j2* | *Cox4i1* | *Eif4h* | *Hsbp1* | *Myl12a* | *Psap* | *Rpl27a* | *Rps4x* | *Tma7* |  |
| *Atp5o* | *Cox5b* | *Eif5a* | *Hsd17b12* | *Myl12b* | *Psma2* | *Rpl28* | *Rps5* | *Tmbim6* |  |
| *B2m* | *Cox6a1* | *Eno1* | *Hsp90aa1* | *Myl6* | *Psma4* | *Rpl3* | *Rps6* | *Tmed9* |  |
| *Bag1* | *Cox6b1* | *Ergic3* | *Hsp90ab1* | *Nars* | *Psma6* | *Rpl30* | *Rps7* | *Tmem50a* |  |
