# Supplementary figures and images for: G-quadruplexes Stabilization Upregulates CCN1 and Accelerates Aging in Cultured Cerebral Endothelial Cells
Source: Front Aging. 2022 Jan 12;2:797562. doi: 10.3389/fragi.2021.797562 (PMC9261356; doi:10.3389/fragi.2021.797562)

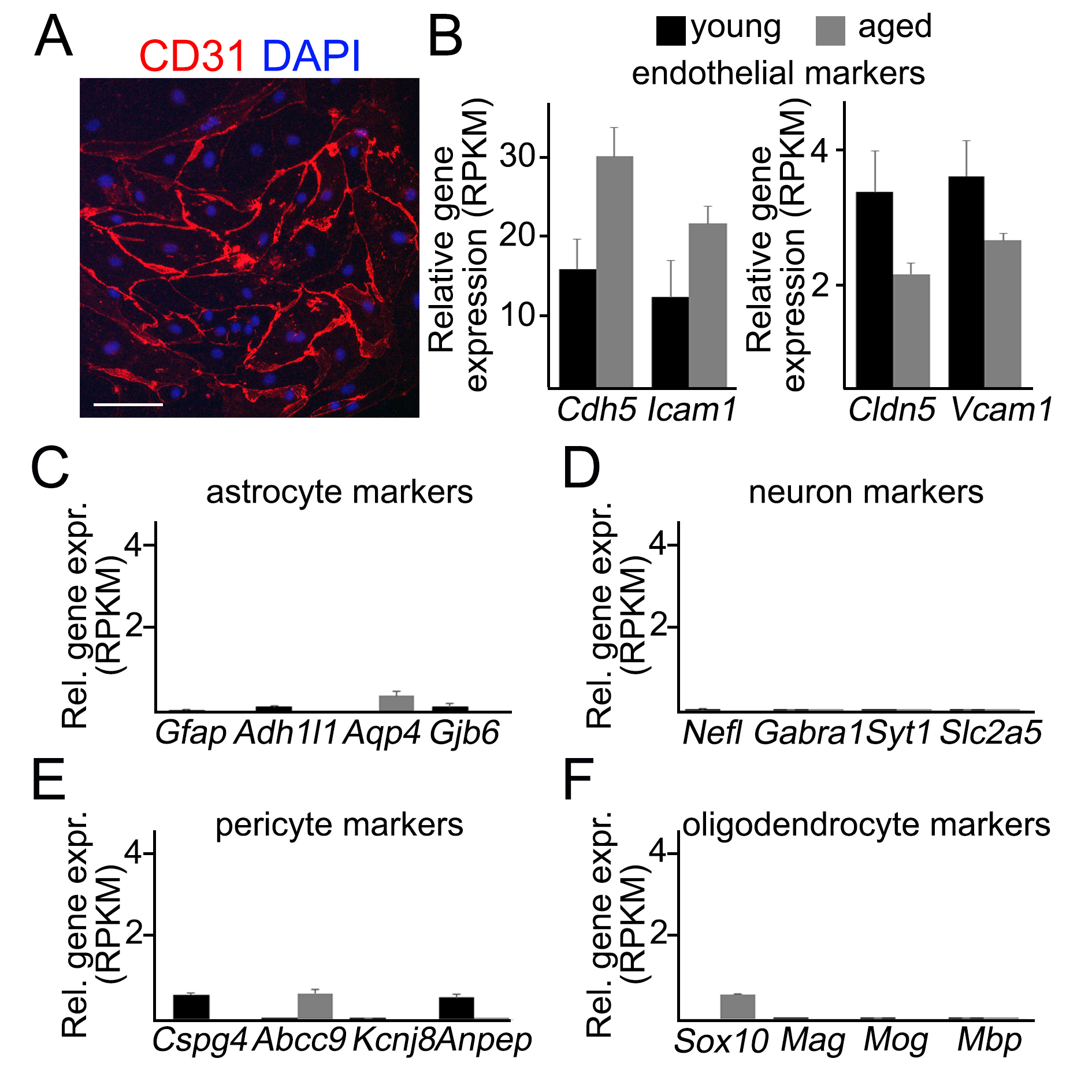

Supplement: Supplementary file 2 [file Image1.JPEG]
